# pH Stability and Antioxidant Power of CycloDOPA and Its Derivatives

**DOI:** 10.3390/molecules23081943

**Published:** 2018-08-03

**Authors:** Shiori Nakagawa, Zetryana Puteri Tachrim, Natsumi Kurokawa, Fumina Ohashi, Yasuko Sakihama, Takeyuki Suzuki, Yasuyuki Hashidoko, Makoto Hashimoto

**Affiliations:** 1 Division of Applied Bioscience, Graduate School of Agriculture, Hokkaido University; Kita 9, Nishi 9, Kita-ku, Sapporo 060-8589, Japan; sh.naka-0408@frontier.hokudai.ac.jp (S.N.); z317_style@live.com (Z.P.T.); natsumi.k0420@gmail.com (N.K.); fumina28ohsei@gmail.com (F.O.); sakihama@abs.agr.hokudai.ac.jp (Y.S.); yasu-h@abs.agr.hokudai.ac.jp (Y.H.); 2 Division of Applied Science, The Institute of Scientific and Industrial Research, Osaka University, Mihogaoka, Ibaraki-shi, Osaka 567-0047, Japan; suzuki-t@sanken.osaka-u.ac.jp

**Keywords:** cycloDOPA, pH stability, antioxidant power

## Abstract

CycloDOPA (leukodopachrome), a well known metabolite of tyrosine, is a precursor of melanine in mammalian organisms and of the pigment betalain in plants. However, the isolation of cycloDOPA from natural sources has not been widely reported. In the present work, the stabilities of cycloDOPA and cycloDOPA methyl ester at various pH levels were studied. Both compounds were stable under acidic conditions. By contrast, both compounds were unstable when the pH was shifted from neutral to basic to form indole derivatives as major products. Based on the pH stability, cycloDOPA and its derivatives were subjected to the DPPH radical scavenging assay for the first time.

## 1. Introduction

CycloDOPA (leukodopachrome), a major metabolite derived from tyrosine, is a precursor of melanin in animals [1] and of betalain pigment in plants [2]. Typically, tyrosine is oxidized by tyrosinase to form DOPA, which is then oxidized to generate dopaquinone and then cyclized to form cycloDOPA. Then, cycloDOPA can undergo either a Schiff base formation reaction or glycosidation, and in the presence of betalamic acid, the formation of betalain (betanin) may subsequently occur [3].

The catechol moiety of cycloDOPA may contribute to the antioxidant properties of the compound, but no detail analysis has been reported yet. The total synthesis of cycloDOPA has been reported in 1968 [4], and is based on the oxidation of catechol, followed by the formation of a five-membered ring with an α-amino moiety tethered to an aromatic ring. Although cycloDOPA is formed following reduction, as outlined in the reaction scheme, the isolation of the cycloDOPA derivative is achieved following triacetylation, and subsequent deacetylation of this isolated derivative is crucial for producing pure cycloDOPA hydrochloride salts. 

In previously conducted biological studies, the formation of cycloDOPA in plants has been determined by indirect methods. In one of the most common methods, in situ-generated cycloDOPA has been derivatized to triacetyl-protected cycloDOPA [5]. Another method has been developed by reacting the in situ-generated cycloDOPA with betalamic acid to form betalain [6], which has a high molar coextinction coefficient. To the best our knowledge, biosynthetic cycloDOPA has been detected by UHPLC­Q­TOF­MS [7], but studies have not reported the isolation and characterization of cycloDOPA from natural sources. These findings indicated that the chemical stability of cycloDOPA may hamper the isolation of pure cycloDOPA, especially in the case of quantitative analysis from enzymatic reactions. This paper details the chemical stability of cycloDOPA and presents the first study on the antioxidant activity of cycloDOPA and its derivatives.

## 2. Results and Discussion

### 2.1. Synthesis

**Figure 1:**
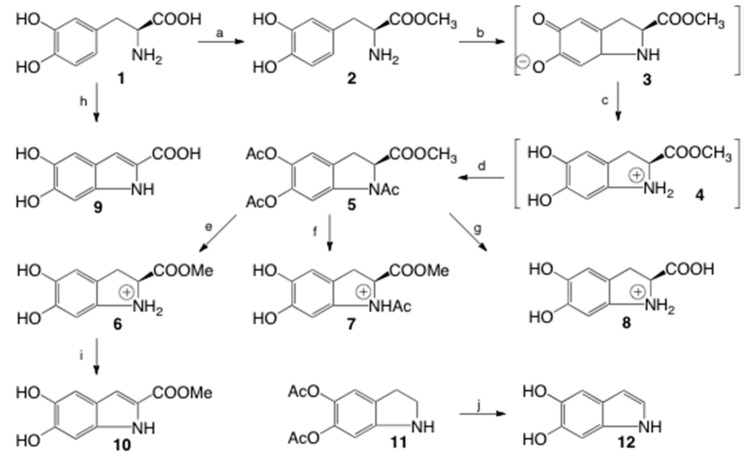
Synthetic scheme for the preparation of cycloDOPA derivatives. (a) SOCl_2_, MeOH, reflux, 1h, quant; (b) K_3_[Fe(CN)_6_], sodium phosphate (pH = 8), 0 °C, 75 s; (c) Na_2_S_2_O_4_, sodium phosphate (pH 8), 0 °C, 30 s; (d) Ac_2_O, pyridine, rt, 4 h, 60% (3 steps); (e) AcCl, MeOH, 60 °C, 16 h, 97%; (f) 1 M HCl, MeOH, rt, 3 days, 59%; (g) 6 M HCl, 80 °C, 5 h, 94%; (h) (1) K_3_[Fe(CN)_6_], NaHCO_3_, rt, 5 min, (2) 1M NaOH, rt, 15 min, 71%; (i) NaOH (pH = 10), rt, o/n, 48%, (j) K_2_CO_3_, MeOH, 0 °C, 50 min, 51%.

Figure 1 shows the synthetic scheme used to produce cycloDOPA and its derivatives. Based on a previous study [4], DOPA­OMe (**2**) was oxidized in the presence of K_3_[Fe(CN)_6_] in a phosphate buffer (pH = 8) followed by immediate reduction with Na_2_S_2_O_4_ to form cycloDOPA­OMe (**3**). The generated cycloDOPA-OMe, which cannot be isolated at this stage, was protected via the formation of triacetyl cycloDOPA-OMe (**5**) in acetic anhydride and pyridine, affording 5 in an isolated yield of 60%. This yield was dramatically different from that reported in the study upon which this synthetic method was based (reported yield of 83%, Supplementary Materials SM-1), but it was in agreement with a more recent study that used the same reaction scheme [8] (reported yield of 64%). Partial and full deprotection of compound **5** was achieved as follows. Treatment with acetyl chloride in methanol at 60 °C for 16 h afforded cycloDOPA-OMe (**6**). The reaction of compound **5** with 1 M HCl in methanol at room temperature for 3 days afforded *N*-Ac cycloDOPA-OMe (**7**). Fully deprotection for 5 was achieved with 6 M HCl at 80 °C for 5 h and afforded cycloDOPA (**8**). The synthetic compounds were identical with NMR analysis (Supplementary Materials SM-2). Reaction protocols indicated that this cycloDOPA skeleton might be unstable under alkaline conditions and stable under acidic conditions.

Compound **8**, which is generated in situ from tyrosine or DOPA oxidation, has been reported to be metabolized into 5,6-dihydroxyindole (DHI, **12**) and 5,6-dihydroxyindole-2-caroxydehydroxyindole (DHICA, **9**) [2]. Therefore, cycloDOPA metabolites were also synthesized to comprehensively analyze the chemical stability of cycloDOPA. DHI was synthesized from commercially available diacetyloxy indole using alkaline treatment in a moderate yield, and the successful synthesis of this compound was verified by ^1^H-NMR and 2D NMR in acetone-d_6_, and H3 exhibits an upfield chemical shift of 6.2 ppm [9]. DHICA was synthesized from 1 by oxidation with K_3_[Fe(CN)_6_] in the presence of sodium bicarbonate, followed by alkaline treatment [10]. All proton signals appeared as singlets, and detailed 2D NMR analysis clearly identified the source of each signal. DHICA-OMe was isolated in 48% yield from the alkaline treatment (pH = 10) of **6**.

### 2.2. Analysis of The pH Stability of CycloDOPA (***8***) and CycloDOPA Methyl Ester (***6***)

**Figure 2:**
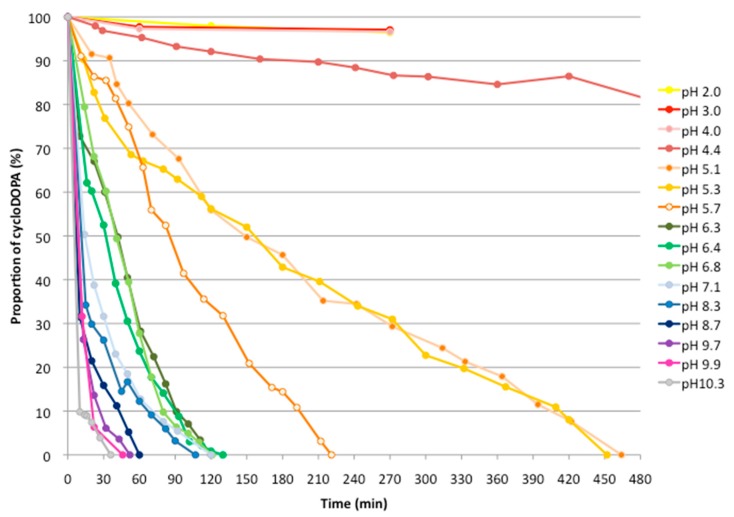
pH stability of cycloDOPA (**8**) in D_2_O solution. Solutions of CycloDOPA (4 mg) in D_2_O (0.5 mL, 34 mM, pH < 2) were adjusted to pH levels ranging from 2 to 10 using 1 M NaOD/D_2_O. The resulting samples were analyzed, and the decreases in the α- and β- protons of cycloDOPA were calculated.

Enzymatically generated cycloDOPA was stable after 8 min at 30 °C, but decomposed after 30 min at the same temperature at a neutral pH = 8. Details related to the stability of this compound, however, have not been clarified under various pH conditions [6]. The pH of a D_2_O solution of cycloDOPA (35 mM) was adjusted using 1 M NaOD/D_2_O from a pH of 2 to 10.3, and the resulting samples were subjected to ^1^H-NMR analysis after an allotted amount of time had elapsed. The characteristic decrease in the observed amounts of the α- and β- protons helped to identify the decomposition of this compound under these conditions (Supplementary Materials SM-3). Figure 2 shows the results obtained from these experiments.

CycloDOPA was found to be stable at a pH of less than 4. The decomposition of cycloDOPA started at pH = 4.5, and complete decomposition was observed after 8 h at a pH = 5. At neutral pH, cycloDOPA decomposed within 2 h. These results are in agreement with those obtained previously, in which the enzymatic reaction mixture was incubated for a longer period (>0.5 h), and the formation of betalain was not observed [6]. The decomposition of cycloDOPA was promoted at higher pH values, and it was completely decomposed within 1 h a pH of greater 8.7.

**Figure 3:**
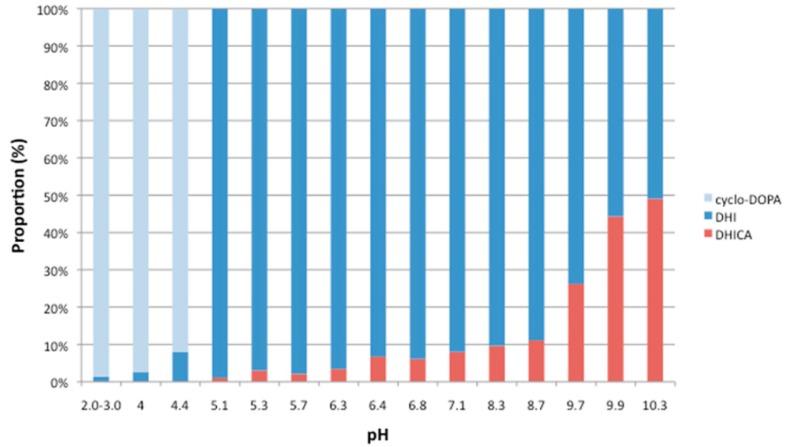
The products distibutions of the decomposition of cycloDOPA (**8**) at different pH levels. CycloDOPA (**8**, 35 mg) was dissolved in D_2_O (0.5 mL) and CH_3_CN (1 μL), and the solution was adjusted to the indicated pH with a NaOD solution. The incubation time refers to the time required for cycloDOPA to be consumed completely, expected pH = 3.6 and pH = 4.4.

Next, the analysis of the end products in the treated cycloDOPA mixture at various pH values were carried out by ^1^H-NMR. DHI and DHICA were detected as main products by ^1^H-NMR analysis, and specific splitting patterns for the analysis of each compound were observed. The signal at the H3 position of DHI was detected at 6.2 ppm, and the signals of the H3 and H7 protons of DHICA were detected at 6.9 ppm as two singlets (see Supplementary Materials SM-3). At pH levels above 9.6, DHI (**12**) was detected as the main product from the decomposition of cycloDOPA. On the other hand, similar amounts of DHICA and DHI were detected at a pH of 10.3 after 40 min. Figure 3 shows the analysis of cycloDOPA (**8**), DHI (**12**), and DHICA (**9**) at each pH.

These results indicate that the molecular form of cycloDOPA (**8**) is stable at pH levels less than 4, and it is converted to an indole by decarboxylation to produce DHI (**12**) via dopachrome at pH values ranging from 4 to 9. Higher pH values prompted the generation of the salt form of the carboxylic acid moiety and inhibited decarboxylation such that the amount of 9 was increased. In the oxygen-free condition, half of cycloDOPA was converted to DHI (**12**) within several minutes and the amounts of both compounds were unchangeable until 90 min at pH = 10.

**Figure 4:**
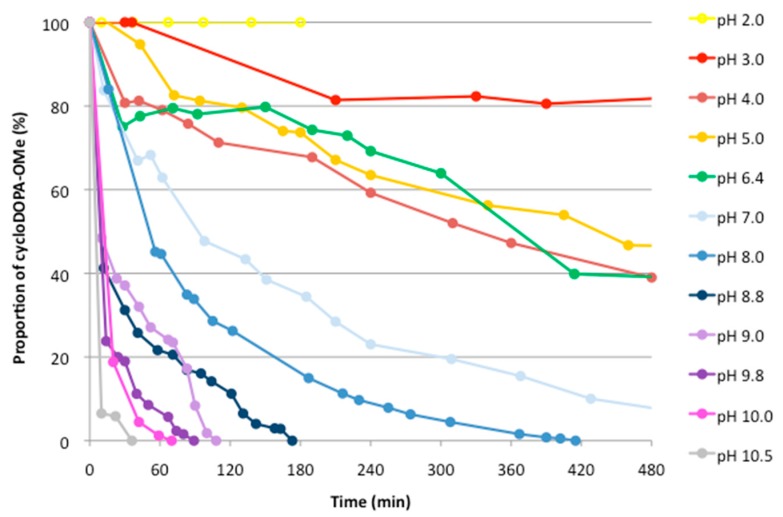
pH stability of cycloDOPA-OMe (**6**) in a D_2_O solution. Solutions of cycloDOPA methyl ester (4 mg) in D_2_O (0.5 mL, 34 mM, pH < 2) were adjusted to each of the listed pH values using 1 M NaOD/D_2_O. These samples were measured after certain time intervals, and the decrease in the α- and β- protons of the cycloDOPA-OMe was calculated.

The formation of carboxylic acid may have affected the formation of the indole from the indoline skeleton. In addition, cycloDOPA-OMe (**6**) was subjected to pH stability experiments (Figure 4). CycloDOPA-OMe (**6**) (33 mM) in a D_2_O solution was adjusted to pH values ranging from 2 to 10.5 using NaOD and subjected to ^1^H-NMR analysis. 

The results indicate that 40% of cycloDOPA-OMe (**6**) remained in the reaction mixture after 8 h at pH = 6.4, and lower pH levels promote the complete decomposition of 8 within 2 h. Higher pH values (pH > 9), however, promoted the decomposition of cycloDOPA-OMe (**6**) within 2 h. The decomposed product was identified as DHICA-OMe (**10**) (Figure 4).

### 2.3. DPPH Assay for CycloDOPA and Its Related Products

To date, the antioxidant activity of cycloDOPA (**8**) has not been reported because of its chemical instability at neutral pH. The findings obtained herein with respect to the chemical stability of cycloDOPA in relation to pH revealed that cycloDOPA can be subjected to the DPPH assay [11] at different pH values, because at a pH of 4, it was determined to be stable, and at a pH of 6, it was determined to be only slightly decomposed (less than 30%) within the assay period (20 min). 

**Figure 5:**
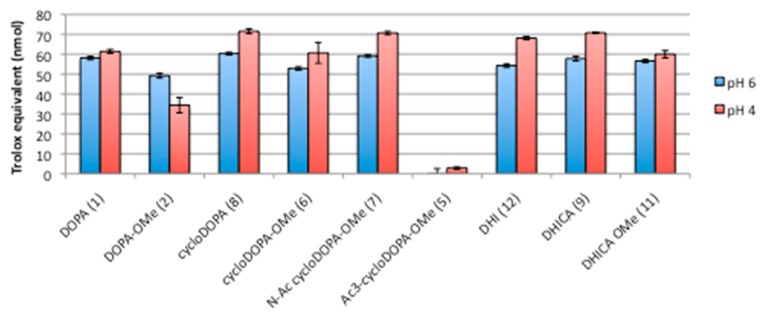
DPPH radical scavenging activity of cycloDOPA and its derivatives. Samples (20 μM) were subjected to the scavenging assay after an incubation time of 20 min. Activities were expressed by Trolox equivalent values. The DPPH assay was performed in triplicate for each sample.

Based on the methods reported by Takebayashi [12], which were used to conduct the measurements at low pH levels, cycloDOPA (**8**) and its derivatives (1, 2, 5, 6, 7, 9, 10, and 12) were subjected to the DPPH assay. At pH values of 4 and 6, the DPPH radical scavenging activities of the compounds, with the exception of triacetyl-cycloDOPA-OMe (**5**), were dependent on their respective concentrations (Supplementary Materials SM-4). Among the tested compounds, cycloDOPA (**8**) exhibited the highest DPPH radical scavenging activity at a low concentration (8 μM). Cyclization of the DOPA skeleton and the presence of a carboxylic acid moiety, not methyl ester pretection, enhanced the antioxidant activities of 1, 2, 8, and 6 at pH values of 4 and 6. The metabolites of cycloDOPA, and indole derivatives 12 and 9, were observed to express antioxidant activity because of the *o*-catechol skeleton. In addition, *N*-acetyl-cycloDOPA-OMe (**7**) exhibited antioxidant activity similar to that of other catechol derivatives, indicating that the NH group of the indoline moiety has little influence on the antioxidant activity of this compound. The DPPH radical scavenging activities for the 20 μM samples are presented as Trolox equivalent values in Figure 5.

This is the first instance of detail analysis for the stability of cycloDOPA under various pH conditions. The indoline skeleton of cycloDOPA easily converts the indole skeleton under neutral pH, and the properties promote the instability of the cycloDOPA, which cannot be isolated from a natural source. The catechol moieties of cycloDOPA and its derivatives, not nitrogen atom on indoline and indole skeleton, contribute antioxidant properties. These results will contribute to the analysis for metabolic pathway of tyrosine and DOPA.

## 3. Materials and Methods

### 3.1. General Procedures

All reagents used were analytical grade. NMR spectra were measured by EX 270 spectrometer (JEOL, Tokyo, Japan). Optical rotations were measured at 23 °C on JASCO DIP370 polarimeter (JASCO, Tokyo, Japan). HRMS-ESI spectra were obtained with a Waters UPLC ESI-TOF mass spectrometer (Waters, Milford, UT, USA).

### 3.2. Synthesis

*l-DOPA methyl ester hydrochloride* (**2**, DOPA-OMe). SOCl_2_ (5.0 mL, 70 mmol) was added slowly to dry MeOH (20 mL) at −5 °C. l-DOPA (**1**) (1.00 g, 50 mmol) was added to the reaction mixture. The reaction mixture was stirred at rt for 1 h and refluxed at 85 °C for 1 h. After the reaction, the reaction mixture was concentrated to afford a colorless amorphous mass (1.24 g, 100%). ^1^H-NMR (270 MHz, CD_3_OD) δppm: 6.75 (d, *J* = 8.2 Hz, 1H), 6.66 (d, *J* = 2.0 Hz, 1H), 6.55 (dd, 1 H, *J* = 8.1, 2.1 Hz, 1H), 4.21 (dd, *J* = 7.4, 5.8 Hz, 1H), 3.82 (s, 3H), 3.11 (dd, *J* = 14.5, 5.9 Hz, 1H), 3.00 (dd, *J* = 14.5, 7.3 Hz, 1H); ^13^C-NMR (68 MHz, CD_3_OD) δppm: 170.4, 146.7, 146.1, 123.3, 121.9, 117.3, 116.9, 55.4, 53.6, 36.7; HRMS (ESI) *m/z* [M + H]^+^ calcd. for C_10_H_14_NO_4_ 212.0923, found 212.0928; [α]_D_ = + 12 (c 1.0, CH_3_OH); UV (CH_3_OH): λ_max_ (ε) = 283 (2788).

*O,O,N­Triacetyl cycloDOPA methyl ester* (**5**). To a chilled solution of DOPA-OMe hydrochloride (**2**) (800 mg, 3.23 mmol) in 88 mM phosphate buffer (400 mL; pH = 8, prepared by mixing 0.4 g of KH_2_PO_4_ and 9.57 g of Na_2_HPO_4_ in 800 mL water), a solution of K_3_[Fe(CN)_6_] (6.48 g, 19.68 mmol) in phosphate buffer (200 mL) was added at 4 °C. After 8 s, a solution of Na_2_S_2_O_4_ (5.12 g, 29.41 mmol) in phosphate buffer (100 mL) at 4 °C was added, and the mixture was stirred for 30 s. The reaction mixture was adjusted to pH = 1 with concentrated HCl, and then the solution was concentrated and co-evaporated with toluene several times. The residue was suspended in Ac_2_O (40 mL) and pyridine (40 mL). The suspension was stirred at room temperature for 4 h and then filtered through Celite. The insoluble material was washed with CH_2_Cl_2_, and the filtrate was concentrated. The residue was partitioned between CH_2_Cl_2_ (80 mL) and 1 M HCl (80 mL). The organic layer was washed with sat. NaHCO_3_, water and brine, dried over MgSO_4_, filtrated, and concentrated. The residue was purified by silica gel column chromatography (CH_2_Cl_2_/MeOH = 80/1) to afford *O,O,N*-triacetyl cycloDOPA methyl ester (**5**) (645.2 mg, 60%) as a colorless amorphous solid. ^1^H-NMR (270 MHz, CDCl_3_) δppm: 8.03 (s, 1H), 7.00–6.92 (m, 1H), 5.13 (d, *J* = 8.2 Hz, 0.2H), 4.94 (d, *J* = 9.6 Hz, 1H), 3.72 (s, 3H), 3.45 (dd, *J* = 16.6, 11.0 Hz, 1H), 3.16 (d, *J* = 16.8 Hz, 1H), 2.33–2.16 (m, 9H); ^13^C-NMR (68 MHz, CDCl_3_) δppm: 171.3, 168.8, 168.4, 168.2, 141.2, 140.6, 138.2, 126.5, 118.8, 112.5, 61.7, 53.0, 33.0, 23.4, 20.6, 20.5; HRMS (ESI) *m/z* [M + H]^+^ calcd. for C_16_H_18_NO_7_ 336.1078, found 336.1085; [α]_D_ = −80 (c 1.0 CHCl_3_); UV (CH_3_OH): λ_max_ (ε) = 294 (4380).

*CycloDOPA hydrochloride* (**8**). Triacetyl cycloDOPA methyl ester (**5**) (134.5 mg, 0.40 mmol) was dissolved in 20% HCl. The reaction mixture was stirred at 80°C for 4 h, and then concentrated. The residue was washed with CH_3_CN to remove excess HCl and afford the product as brown, amorphous solid (88.0 mg, 0.38 mmol, 95%). ^1^H-NMR (270 MHz, D_2_O) δppm: 6.98 (s, 1H), 6.91 (s, 1H), 4.97 (dd, *J* = 9.2, 6.9 Hz, 1H), 3.58 (dd, *J* = 16.3, 9.7 Hz, 1H), 3.36 (dd, *J* = 16.3, 6.8 Hz, 1H); ^13^C- NMR (68 MHz, D_2_O) δppm: 172.4, 146.8, 145.1, 127.0, 126.1, 112.8, 107.2, 61.5, 33.3; HRMS (ESI) *m/z* [M + H]^+^ calcd. for C_9_H_10_NO_4_ 196.0565, found 196.0607; [α]_D_ = −91.4 (c 0.5 H_2_O); UV (0.1 M HCl): λ_max_ (ε) = 285 (4372).

*CycloDOPA methyl ester* (**6**). Acetyl chloride (0.151 mL, 2.14 mmol) was added to MeOH (5.2 mL) in an ice bath, and triacetyl cycloDOPA methyl ester (**5**) (143.1 mg, 0.43 mmol) was added to the solution. The reaction mixture was stirred at 60 °C for 14 h and concentrated to afford the product as a yellow, amorphous solid (101.2 mg, 97%). ^1^H-NMR (270 MHz, D_2_O) δppm: 7.14 (s, 1H), 7.07 (s, 1H), 5.07 (dd, *J* = 9.2, 7.3 Hz, 1H), 4.04 (s, 3H), 3.74 (dd, *J* = 16.2, 9.2 Hz, 1H), 3.54 (dd, *J* = 16.5, 6.9 Hz, 1H); ^13^C-NMR (68 MHz, D_2_O) δppm: 173.5, 146.8, 145.2, 127.2, 112.9, 107.0, 61.2, 54.6, 33.0; HRMS (ESI) *m/z* [M + H]^+^ calcd. for C_10_H_12_NO_4_ 210.0761, found 210.0733; [α]_D_ = −89 (c 0.7 CH_3_OH); UV (0.1 mM HCl): λ_max_ (ε)= 287 (5676).

*N-acetyl cycloDOPA methyl ester* (**7**). Triacetyl cycloDOPA-OMe (**5**) (76.2 mg, 0.227 mmol) was dissolved in 1 M HCl/MeOH = 1/1 (7.62 mL) and stirred at room temperature for 3 days. After oncentration, the residue was purified by silica gel column chromatography (CH_2_Cl_2_/MeOH = 20/1) to afford N-acetyl cyclo-DOPA methyl ester (**7**) (33.8 mg, 59.2%) as colorless amorphous mass. ^1^H-NMR (270 MHz, CD_3_OD) δppm: 7.67 (s, 1H), 6.82 (s, 0.4H), 6.67 (s, 0.4 H), 6.62 (s, 1H), 5.13 (dd, *J* = 10.7, 2.5 Hz, 1H), 5.07 (dd, *J* = 10.7, 3.5 Hz, 0.3H), 3.76 (3 H, s), 3.71 (1 H, s), 3.39 (dd, *J* = 15.3, 10.1 Hz, 0.4 H), 3.11 (dd, *J* = 16.2, 2.6 Hz, 1H), 2.91 (dd, *J* = 16.0, 3.5 Hz, 0.3H), 2.40 (s, 1.2H), 2.11 (s, 3H); ^13^C-NMR (68 MHz, CD_3_OD) δppm: 173.4, 170.9, 145.9, 145.2, 143.5, 143.2, 136.3, 134.6, 122.8, 121.2, 113.4, 112.1, 106.6, 103.9, 63.2; HRMS (ESI): calcd for C_12_H_14_NO_5_ [M^+^ + H] 252.0866, found 252.0856; [α]_D_ = −67 (c 0.5 CH_3_OH).

*5,6-dihydroxy-2-indolylcarboxylic acid* (**9**, DHICA). To a solution of l-DOPA (**1**) (500 mg, 2.5 mmol) in H_2_O (250 mL) was added a solution of K_3_[Fe(CN)_6_] (6.48 g, 19.7 mmol) in 0.5 M NaHCO_3_ aq (30 mL), and the solution was stirred for 5 min. NaOH (35 mL. 1 M) was added, and the solution was stirred for 15 min. HCl (10 mL, 6 M) was then added to the reaction mixture to adjust the value of pH to 1. The reaction mixture was extracted with EtOAc (3 × 125 mL). The organic layer was washed with brine containing Na_2_S_2_O_5_ (0.095 g) and brine, dried over MgSO_4_, filtrated, and concentrated. The residue was suspended in n-hexane. After the insoluble material was removed by filtration, the supernatant was evaporated to afford 5,6-dihydroxy-2-indolylcarboxylic acid (**9**) (350.4 mg, 72%) as a colorless, amorphous solid. ^1^H-NMR (270 MHz, acetone-d_6_) δppm: 10.37 (s, 1H), 7.05 (s, 1H), 7.00 (s, 1H), 6.98 (s, 1H); ^13^C-NMR (68 MHz, acetone-d_6_) δppm: 163.0, 146.9, 142.6, 134.0, 126.8, 112.7, 108.7, 105.8, 97.6; HRMS (ESI) *m/z* [M + H]^+^ calcd. C_9_H_8_NO_4_ 194.0448, found 194.0447; UV (CH_3_OH): λ_max_ (ε) = 320 (22400).

*5,6-Dihydroxyindole-2-carboxylic acid methyl ester* (**10**, DHICA-OMe). Cyclo-DOPA methyl ester (**6**) (85.4 mg, 0.35 mmol) was dissolved in H_2_O (1.4 mL) and adjusted to pH = 9 with 1 M NaOH. The reaction mixture was stirred at room temperature overnight to generate a precipitate. After the precipitate was filtered off, the filtrated afforded additional precipitates. The precipitates were dried under reduced pressure to afford 5,6-dihydroxyindole-2-carboxylic acid methyl ester (**10**) (34.7 mg, 49%) as a colorless solid. ^1^H-NMR (270 MHz, CD_3_OD) δppm: 6.96 (s, 1H), 6.94 (s, 1H), 6.83 (s, 1H), 3.86 (s, 3H); ^13^C-NMR (68 MHz, CD_3_OD) δppm: 164.1, 147.8, 143.2, 134.7, 126.3, 122.0, 109.3, 106.0, 97.6, 51.9; HRMS (ESI) *m/z* [M + H]^+^ calcd. for C_10_H_10_NO_4_ 208.0604, found 208.0588.

*5,6-dihydroxyindole* (**12**, DHI). 5,6-Diacetoxyindole (**11**) (50 mg, 0.21 mmol, TCI) was dissolved in minimal MeOH (3 mL). To this solution, was added solid anhydrous K_2_CO_3_ (8.8 mg, 0.06 mmol) in MeOH (1 mL) under N_2_ at 0 °C. The reaction mixture was stirred for 50 min at 0 °C under N_2_ and adjusted to pH = 4 with 1 M HCl (100 μL) and then concentrated under an N_2_ atmosphere. The residue was dissolved in deoxygenated Et_2_O and stirred overnight at room temperature. The insoluble material was removed by filtration, and the filtrate was concentrated to afford 5,6-dihydroxyindole (**12**) (16.4 mg, 54%) as a colorless amorphous solid. ^1^H-NMR (270 MHz, CD_3_OD) δppm: 6.97 (s, *J* = 3.3 Hz, 1H), 6.91 (s, 1H), 6.81 (s, 1H), 6.19 (d, *J* = 3.3 Hz, 1H); ^13^C-NMR (270 MHz, CD_3_OD) δppm: 143.6, 141.3, 132.3, 123.7, 122.5, 105.6, 101.5, 97.9, 80.9; HRMS (ESI) *m/z* [M + H]^+^ calcd. for C_8_H_8_NO_2_ 150.0550, found 150.0486; UV (CH_3_OH): λ_max_ (ε) = 303 (3340).

### 3.3. pH Stability for CycloDOPA Derivatives

Cyclo-DOPA (**8**) or cyclo-DOPA-OMe (**6**) (4 mg, 17 μmol) was diluted by D_2_O (500 μL) and adjusted to the value of pH range from 2 to 10 by 1 M NaOD-D_2_O. CH_3_CN (1 μL) was added to the solution. The sample was subjected to ^1^H-NMR analysis after various reaction times.

### 3.4. Evaluation of The DPPH Radical Scavenging Activity for CycloDOPA Derivatives

Compounds **1**, **2**, **6**, and **8** were dissolved in H_2_O (to obtain a pH of 6) or a 10 mM citric buffer (to achieve a pH of 4). Compounds **5**, **12**, **9**, and Trolox were dissolved in EtOH to concentrations of 10 mM and then diluted with H_2_O (to obtain a pH of 6) or 10 mM citric buffer (to obtain a pH of 4). Compounds **7** and **10** were dissolved in MeOH and diluted with H_2_O (to obtain a pH of 6) or 10 mM citric acid buffer (to obtain a pH of 4). A 200 μL portion of DPPH solution (with a final concentration of 50 μM, which was obtained using either EtOH (for a pH of 6) or EtOH/10 mM citric acid buffer = 60/40 (for a pH of 4)) was mixed with 22.2 μL portions of each of the previously described samples, and final concentrations of 0, 4, 8, 12, 16, 20, 40, 100, and 200 μM were obtained. Sample measurements, which were recorded every 10 s, were carried out after 1 min had elapsed. The DPPH solution was added to each sample at 1 min intervals. Changes in the absorbance at 517 nm were measured with a microplate reader. The percentage inhibition of the radical scavenging activity was calculated as follows:

Inhibition (%) = (Control Absorbance − Sample Absorbance)/Control Absorbance × 100

The value was corrected as the Trolox equivalent.

## Supplementary Materials

The following are available online at http://www.mdpi.com/1420-3049/23/8/1943/s1, (SM-1) Optimize the conditions to synthetsis of triacetyl-cycloDOPA-OMe (**5**), (SM-2) NMR data for synthetic compounds, (SM-3) End-products analysis for decomposition of cycloDOPA (**8**) with ^1^H-NMR, (SM-4) Time course analysis for DPPH radical scavenge activity for cycloDOPA and its derivatives at pH = 4 and pH = 6.
