# Supplementary material for: pH Stability and Antioxidant Power of CycloDOPA and Its Derivatives
Source: Molecules. 2018 Aug 3;23(8):1943. doi: 10.3390/molecules23081943 (PMC6222597; doi:10.3390/molecules23081943)
Supplement: Supplementary file 1 [file molecules-23-01943-s001.pdf]

pH Stability and Antioxidant Power of CycloDOPA and Its Derivatives

Shiori Nakagawa [1], Zetryana Puteri Tachrim [1], Natsumi Kurokawa [1], Fumina Ohashi [1], Yasuko Sakihama [1], Takeyuki Suzuki [2], Yasuyuki Hashidoko [1] and Makoto Hashimoto [1,*]

[1] Division of Applied Bioscience, Graduate School of Agriculture, Hokkaido University; Kita 9, Nishi 9, Kita-ku, Sapporo 060-8589, Japan; sh.naka-0408@frontier.hokudai.ac.jp (S.N.); z317_style@live.com (Z.P.T.); natsumi.k0420@gmail.com (N.K.); fumina28ohsei@gmail.com (F.O.); sakihama@abs.agr.hokudai.ac.jp (Y.S.); yasu-h@abs.agr.hokudai.ac.jp (Y.H.)

[2] Division of Applied Science, The Institute of Scientific and Industrial Research, Osaka University, Mihogaoka, Ibaraki-shi, Osaka 567-0047, Japan; suzuki-t@sanken.osaka-u.ac.jp

* Correspondence: hasimoto@abs.agr.hokudai.ac.jp; Tel./Fax: +81-11-706-3849

Supplementary Material

SM-1) Optimize the conditions to synthetsis of triacetyl-cycloDOPA-OMe (**5**)
SM-2) NMR data for synthetic compounds
SM-3) End-products analysis for decomposition of cycloDOPA (**8**) with ^{1}H-NMR
SM-4) Time course analysis for DPPH radical scavenge activity for cycloDOPA and its derivatives at pH 4 and 6.

SM-1) Optimize the conditions to synthetsis of triacetyl-cycloDOPA-OMe **5**

1-1) Reaction time for acetylation

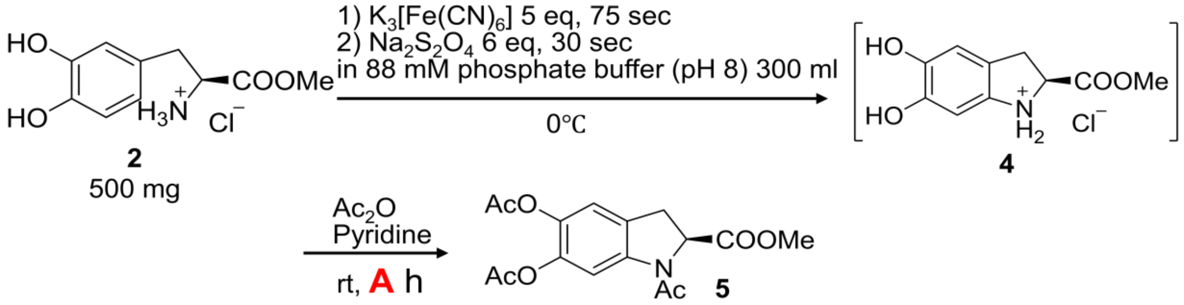

Entry	Reaction time A (h)	Yield of **5**
1	4	28
2	12	21

1-2) Reaction scale

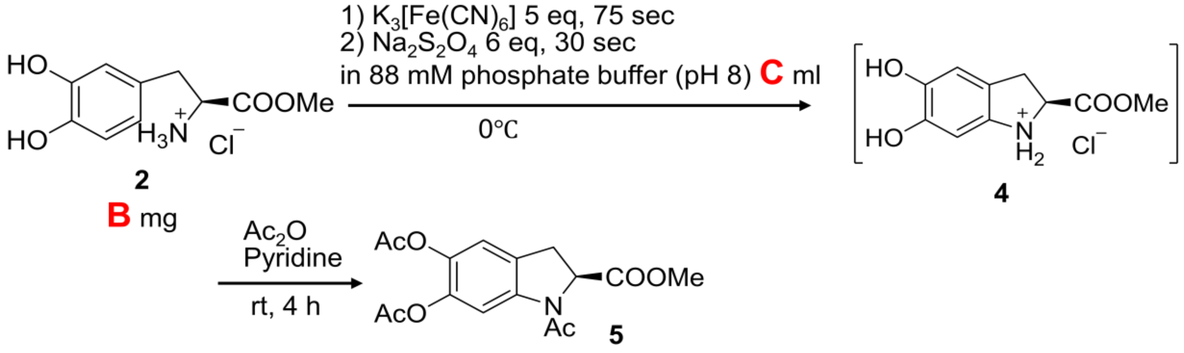

Entry	DOPA-OMe (2) B (mg)	Buffer volume C (ml)	Yield of **5**
1	500	300	28
2	800	480	34
3	1000	600	32
4	1500	900	8

1-3) Reaction time

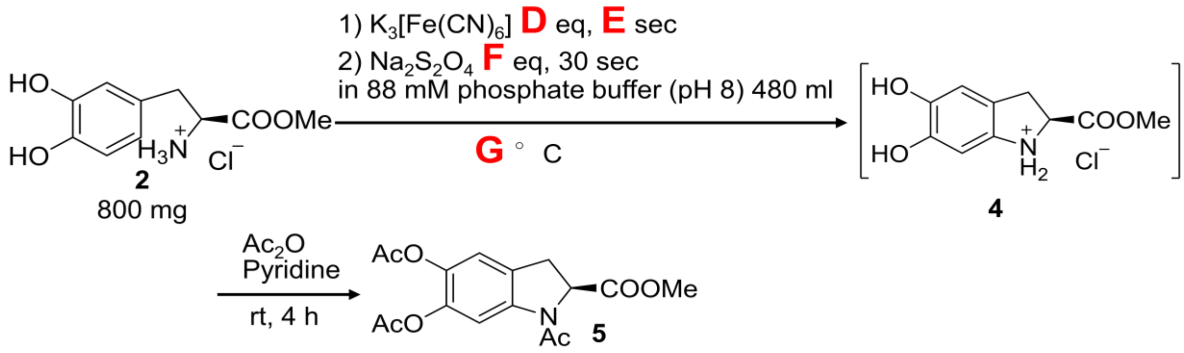

Entry	$K_3[Fe(CN)_6]$ D (eq)	Time E (sec)	$Na_2S_2O_4$ F (eq)	Temp G (°C)	Yield of **5**
1	5	75	6	4	34
2	6.2	15	7.8	rt	41

1-4) Reaction concentration of **2**

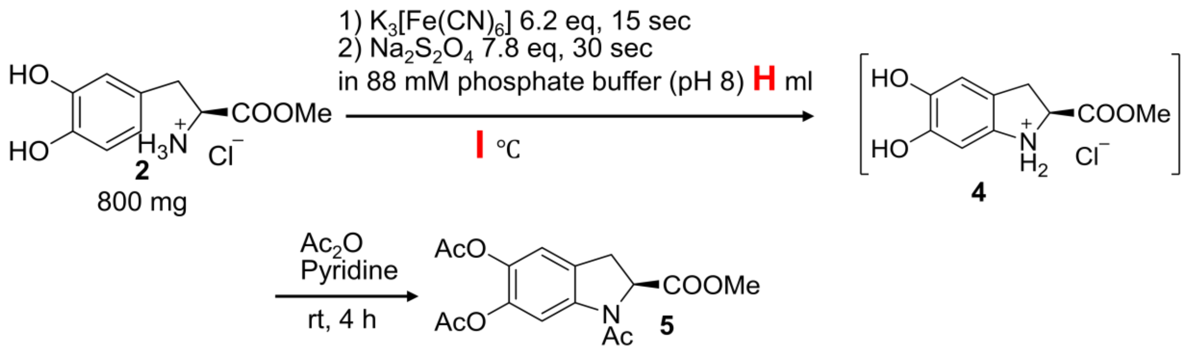

Entry	Buffer volume H (ml)	Temp I (°C)	Yield of **5**
1	480	rt	41
2	120	rt	Complex mixture
3	600	rt	49
4	480	4	52
5	600	4	60

SM-2) NMR data for synthetic compounds

2-1) L-DOPA methyl ester hydrochloride (**2**, DOPA-OMe)

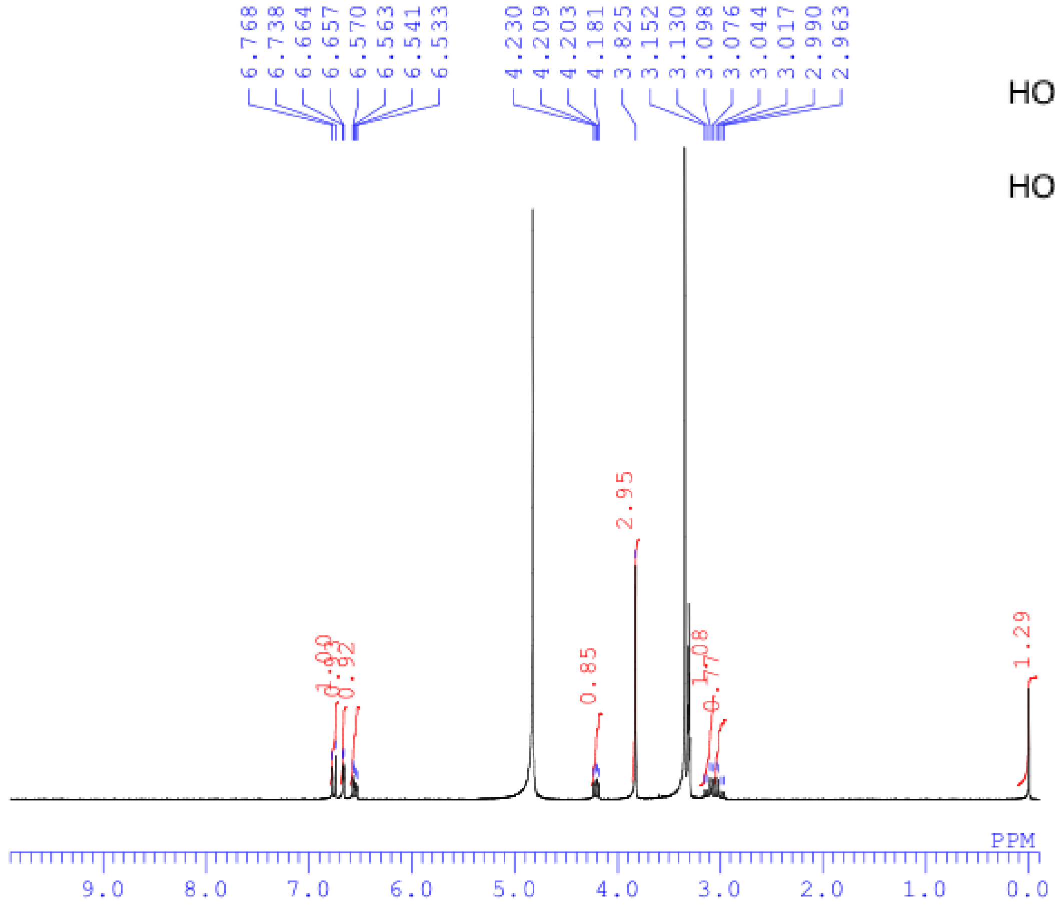

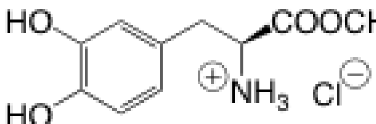

2

(CD_3OD)

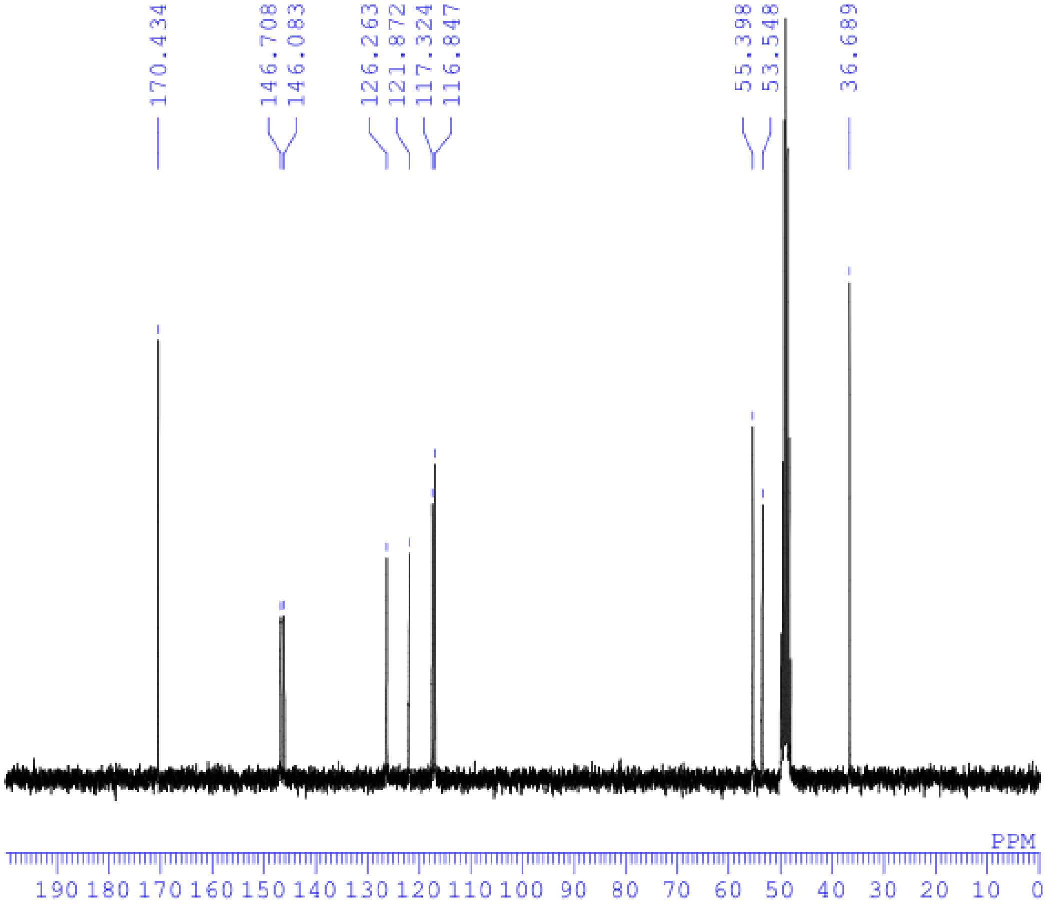

2-2) *O, O, N*- triacetyl cycloDOPA-OMe (**5**)

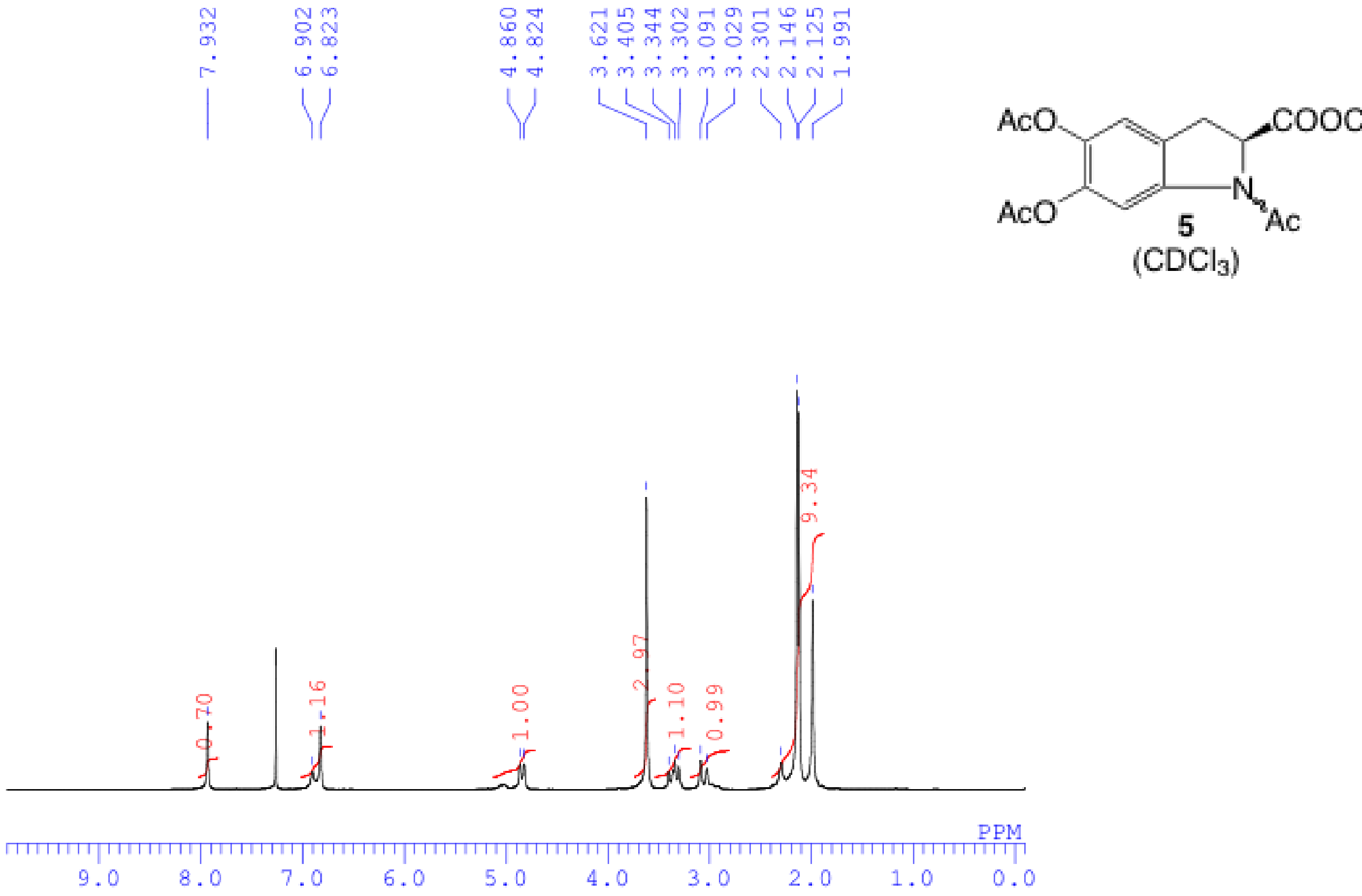

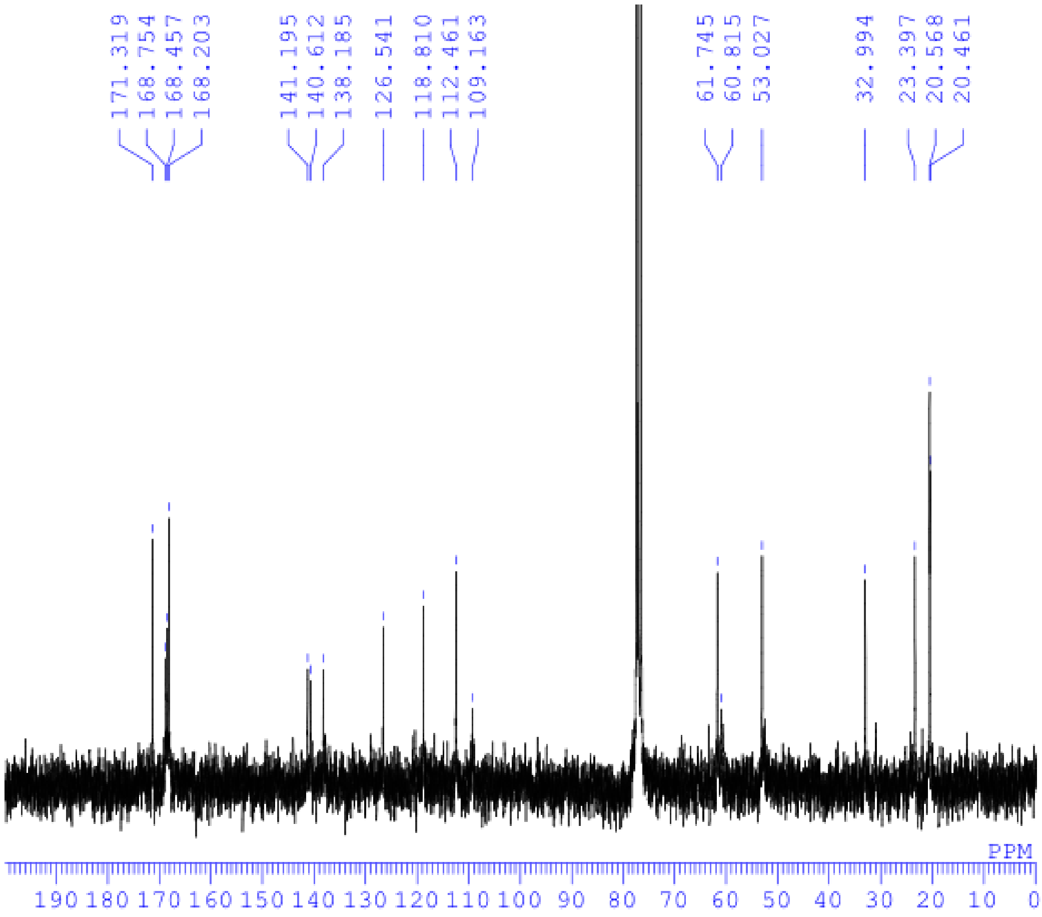

2-3) cycloDOPA-OMe (**6**, cycloDOPA-OMe)

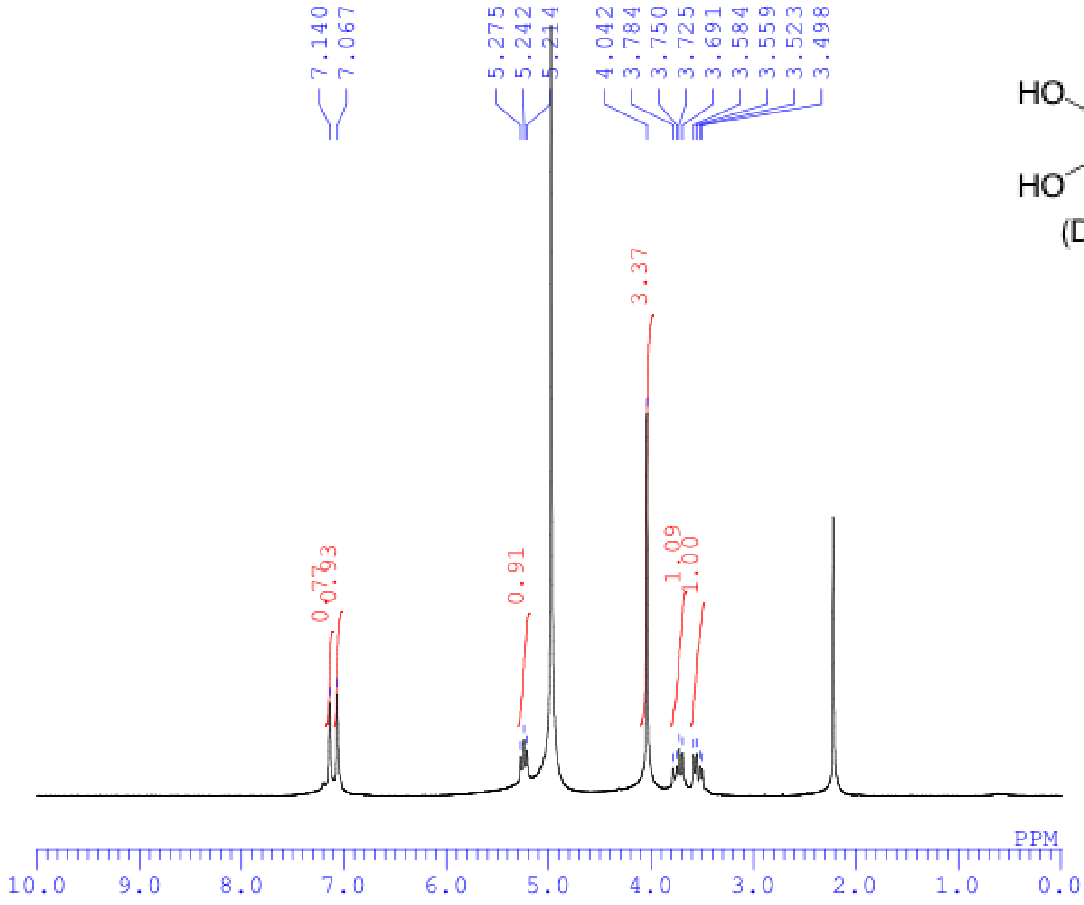

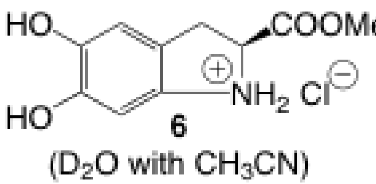

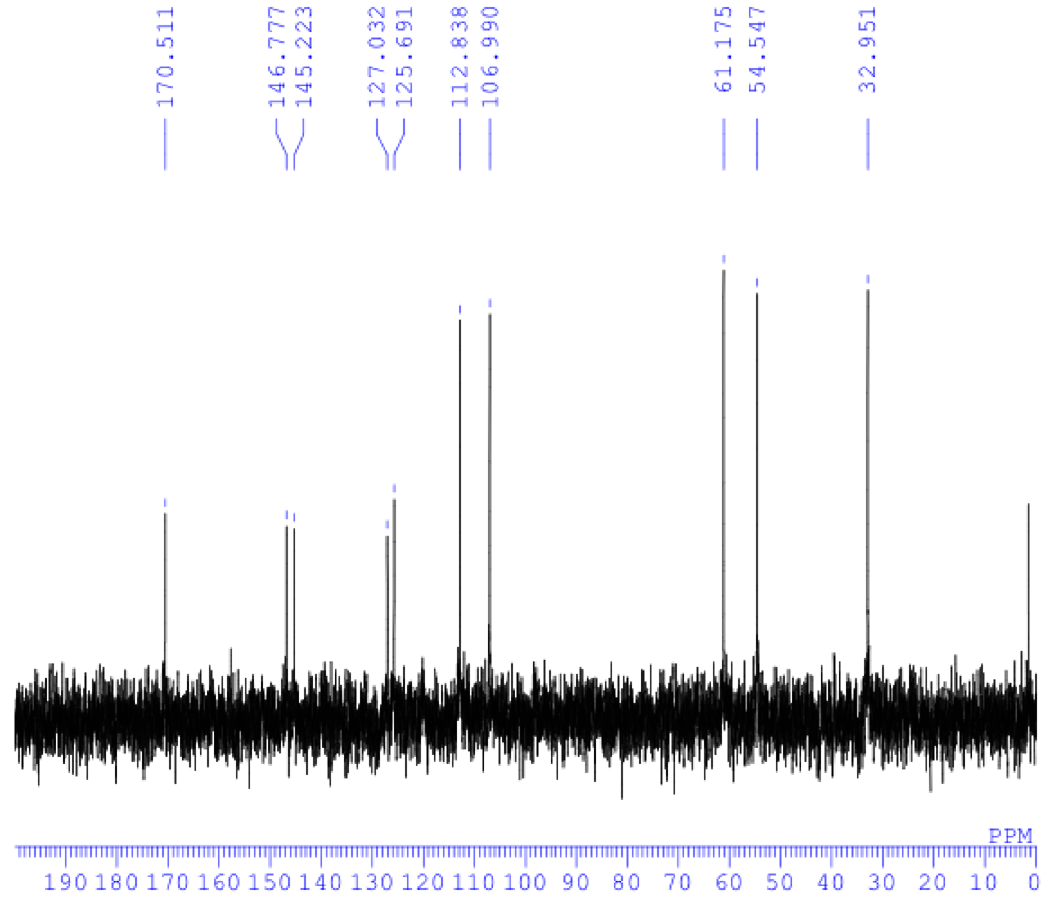

2-4) *N*-acetyl cycloDOPA-OMe (**7**)

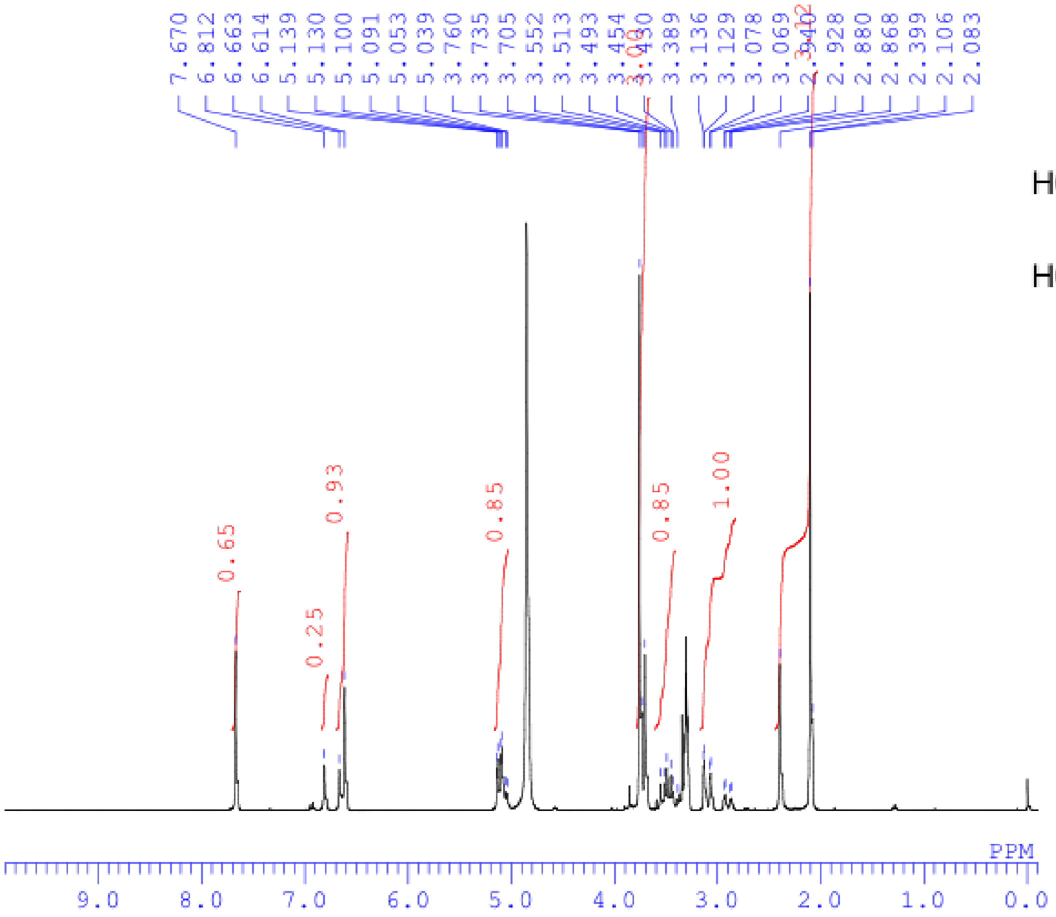
7.670
6.812
6.663
6.614
5.139
5.130
5.100
5.091
5.053
5.039
3.760
3.735
3.705
3.552
3.513
3.493
3.454
3.430
3.389
3.136
3.129
3.078
3.069
2.928
2.880
2.868
2.399
2.106
2.083
0.65
0.25
0.93
0.85
3.00
0.85
1.00
3.02
PPM
9.0
8.0
7.0
6.0
5.0
4.0
3.0
2.0
1.0
0.0

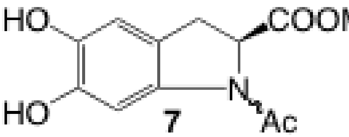
HO
HO
COO
N
Ac

7

(CD_3OD)

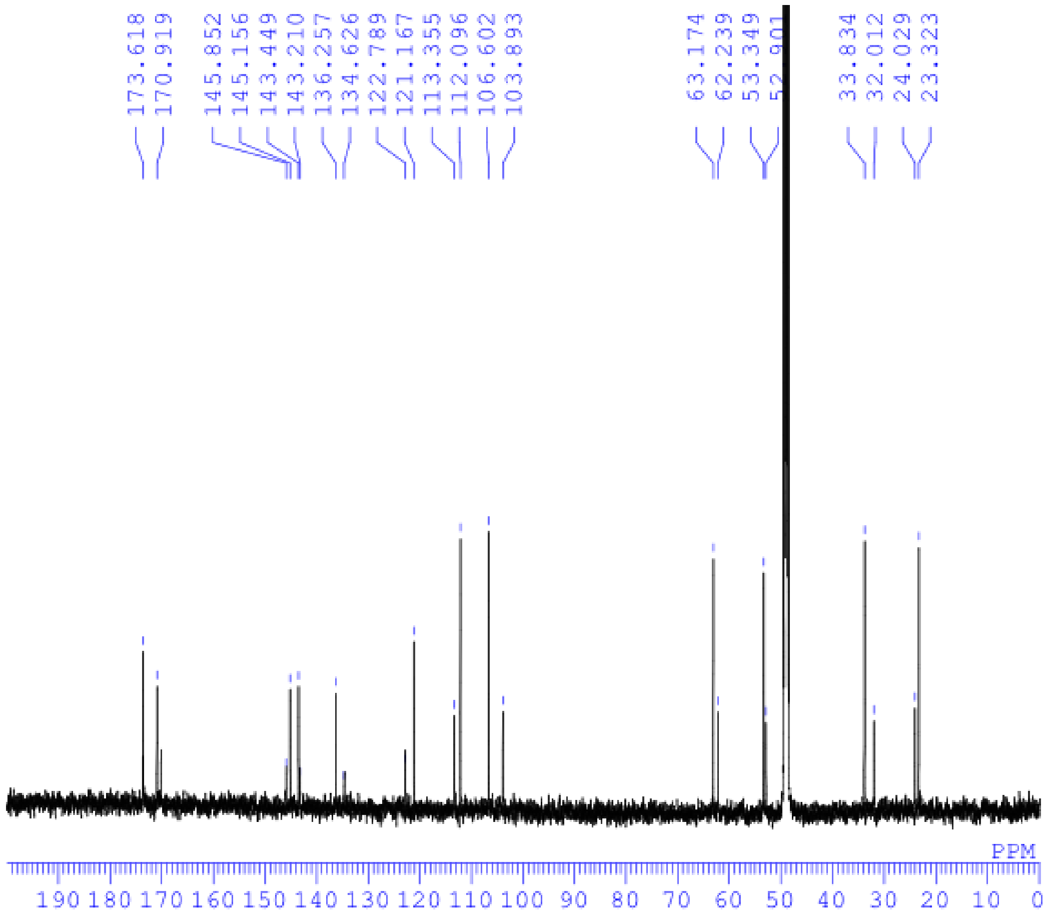
173.618
170.919
145.852
145.156
143.449
143.210
136.257
134.626
122.789
121.167
113.355
112.096
106.602
103.893
63.174
62.239
53.349
52.901
33.834
32.012
24.029
23.323
PPM
190 180 170 160 150 140 130 120 110 100 90 80 70 60 50 40 30 20 10 0

2-5) cycloDOPA hydrochloride (**8**)

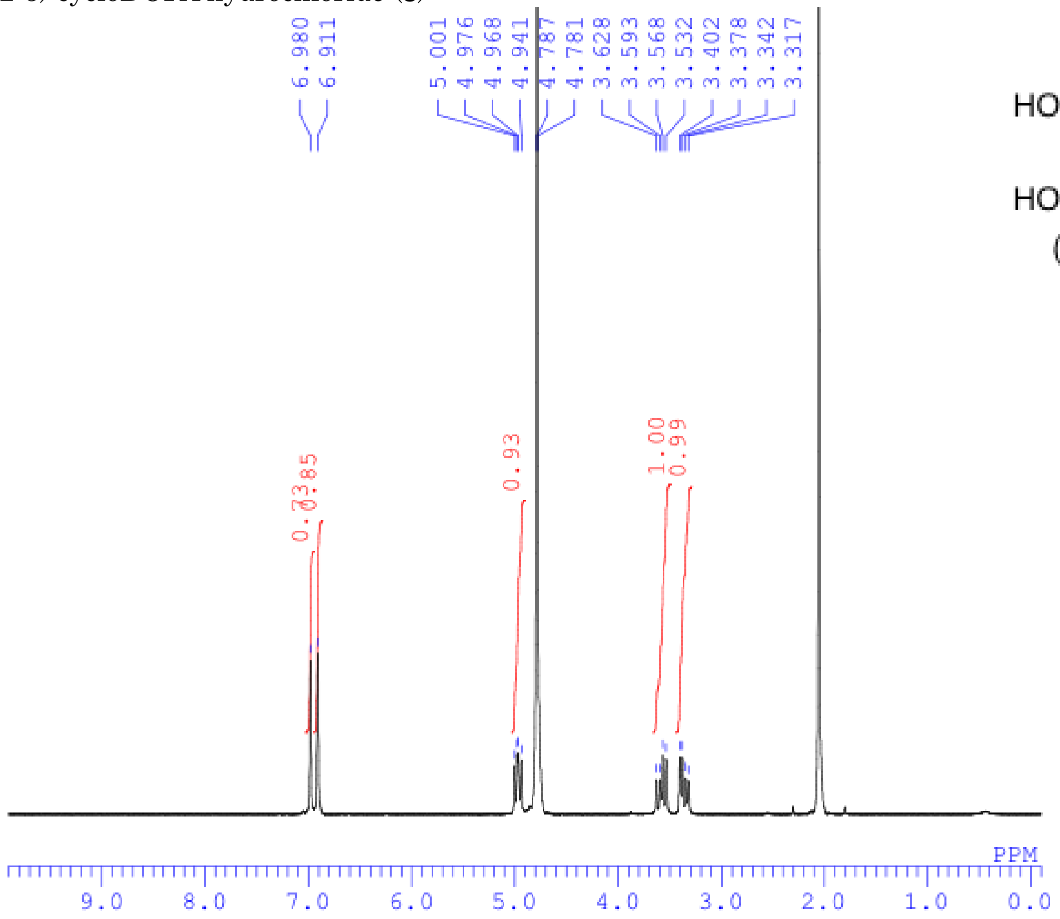

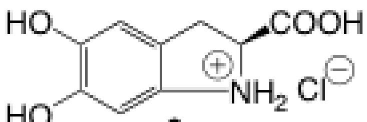

8

(D_2O with CH_3CN)

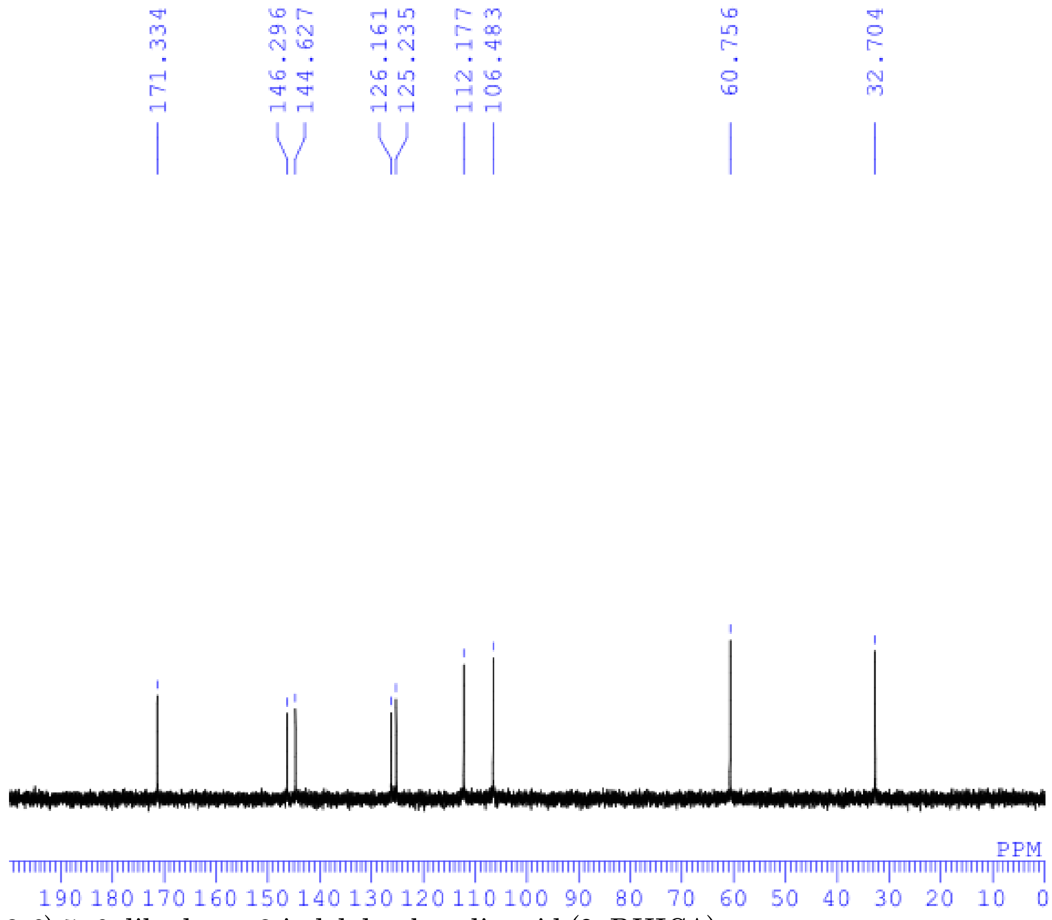

2-6) 5, 6-dihydroxy-2-indolylcarboxylic acid (**9**, DHICA).

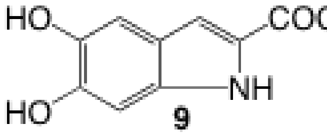

9
(acetone-d_6)

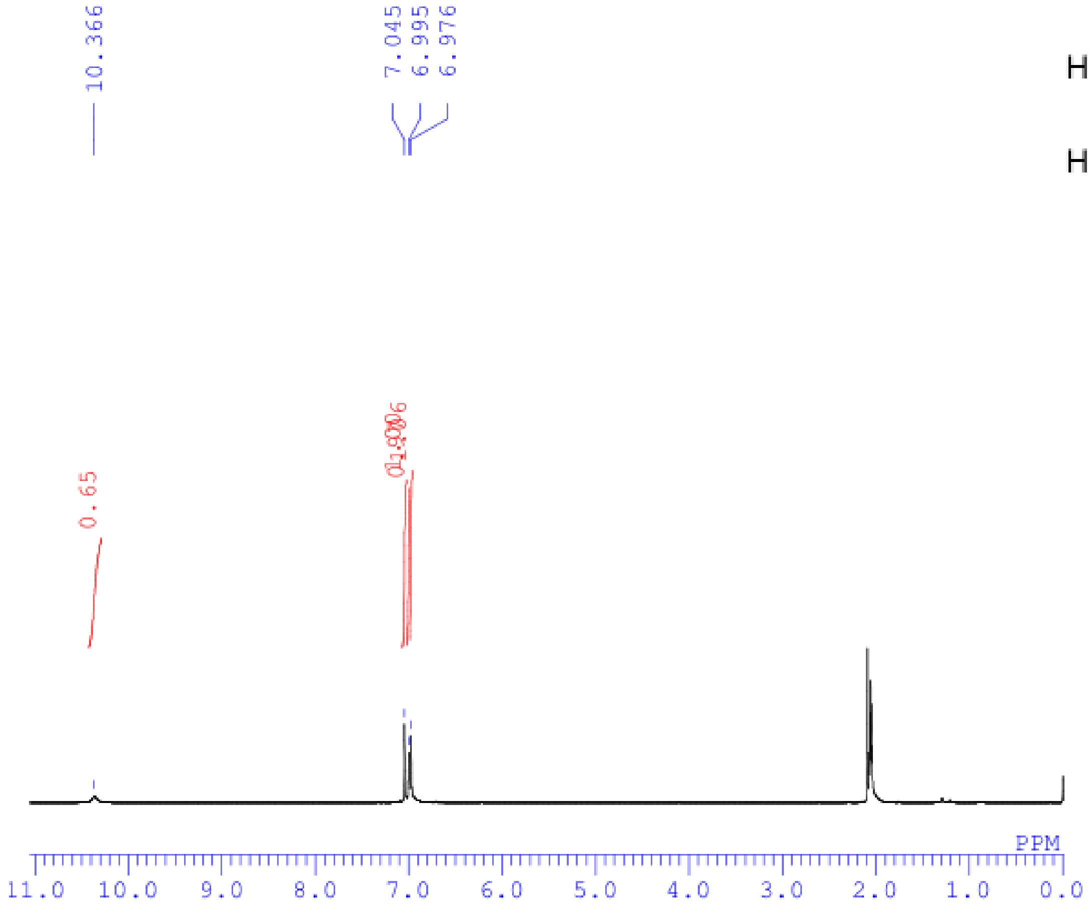

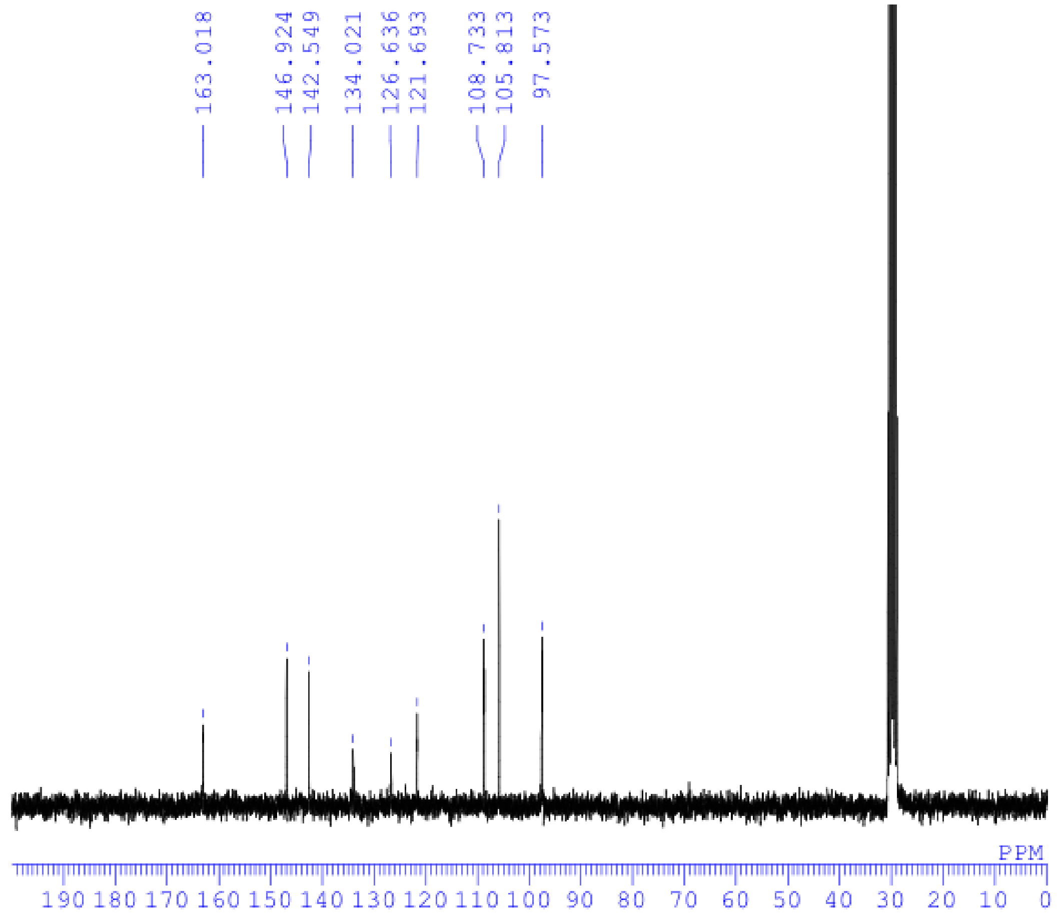

2-7) 5, 6-Dihydroxyindole-2-carboxylic acid methyl ester (**10**, DHICA-OMe)

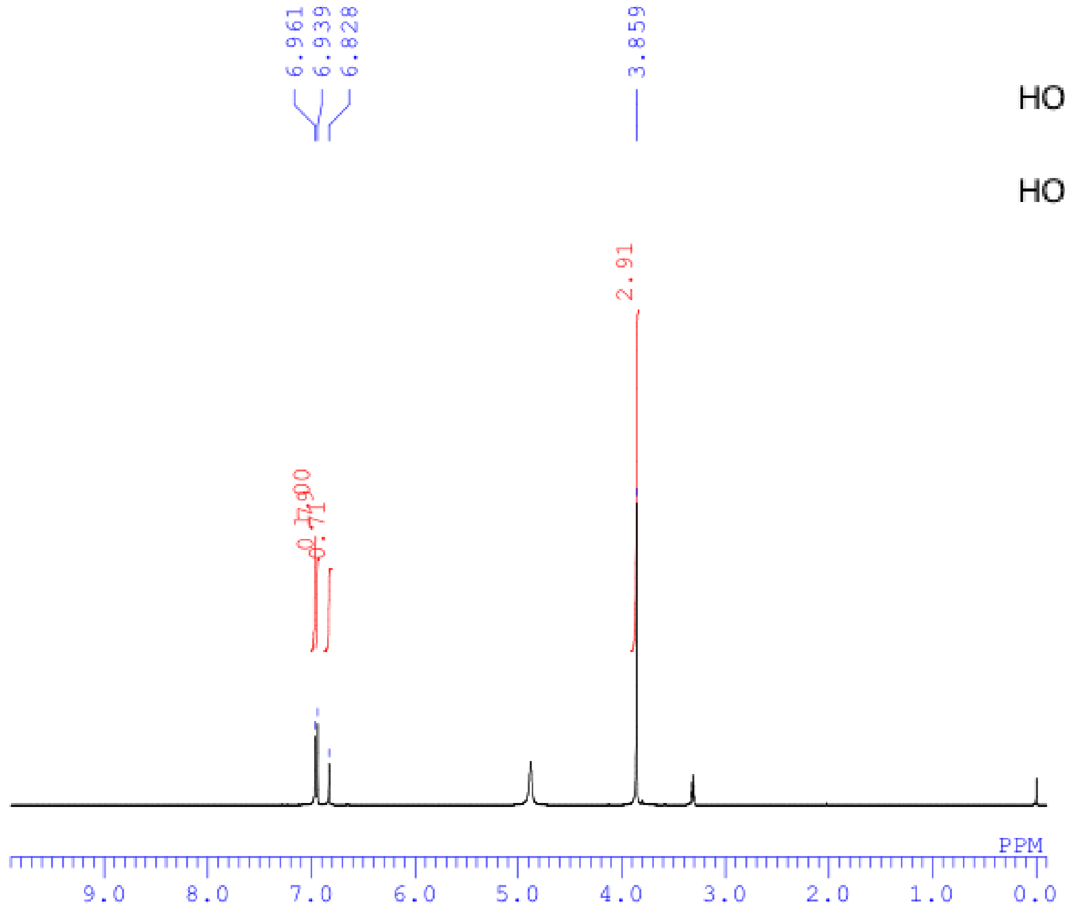

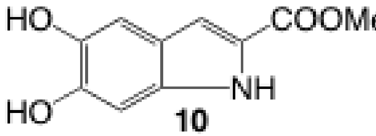

10
(CD_3OD)

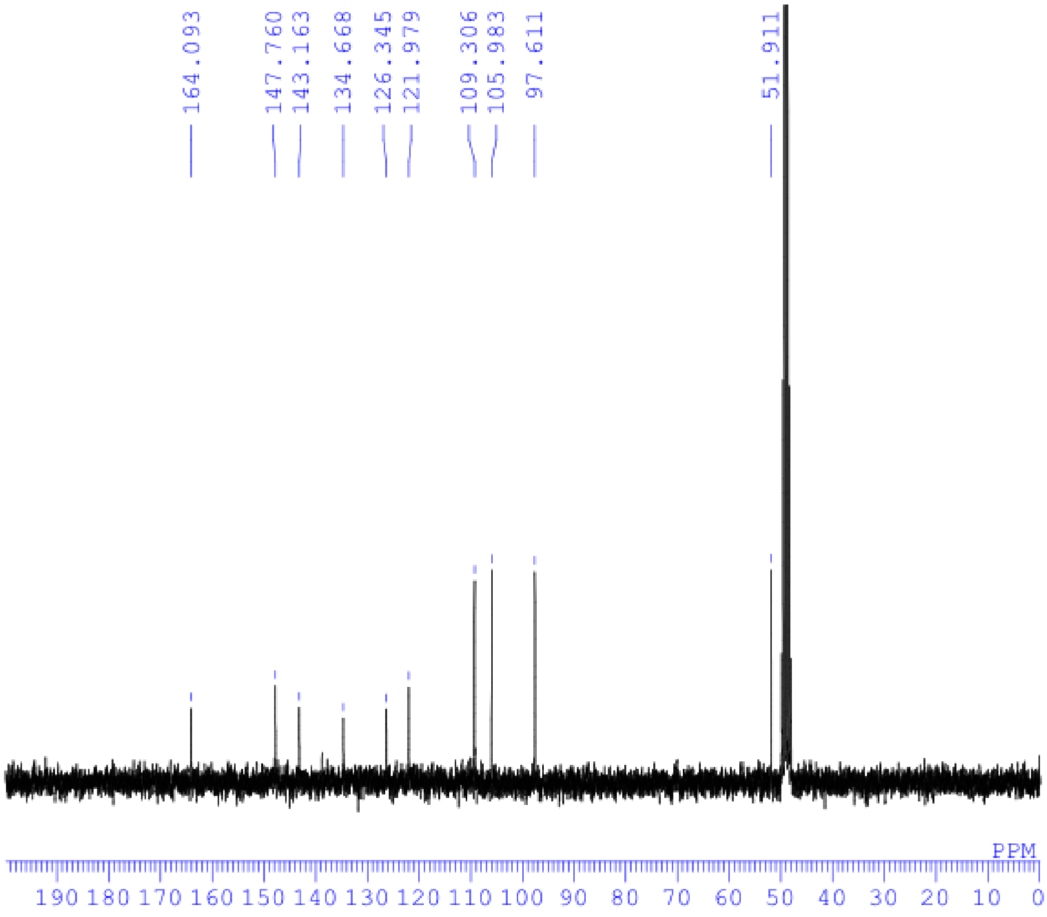

2-8) 5, 6-dihydroxyindole (**12**, DHI)

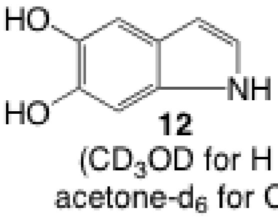

12

(CD_3OD for H

acetone-d_6 for C

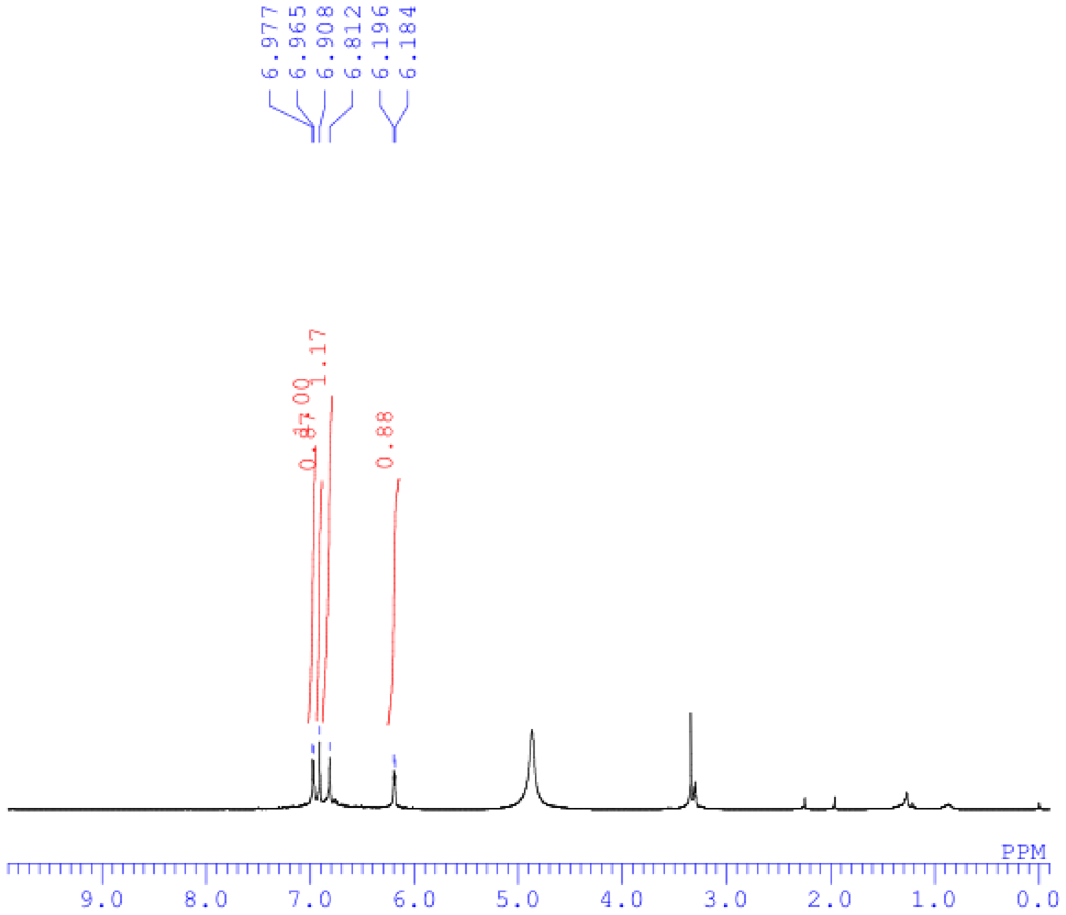

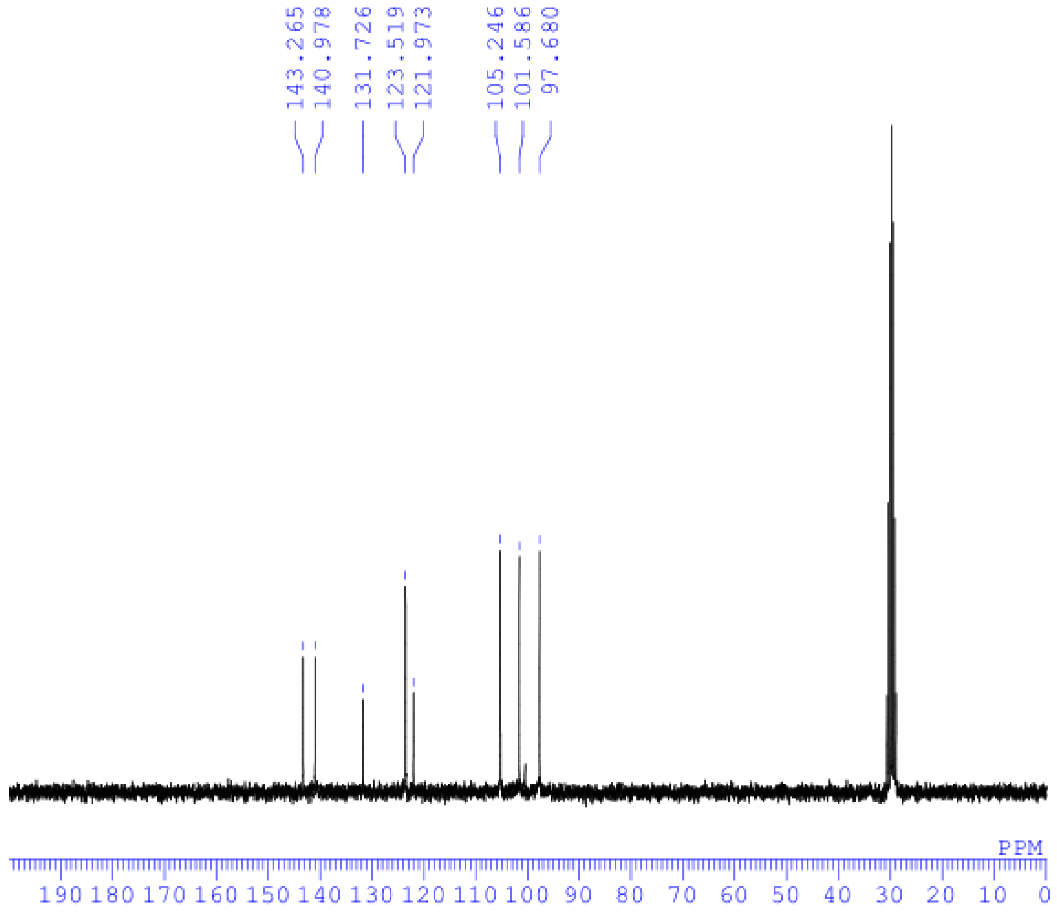

SM-3) End-products analysis for decomposition of cycloDOPA (**8**) with ^{1}H-NMR

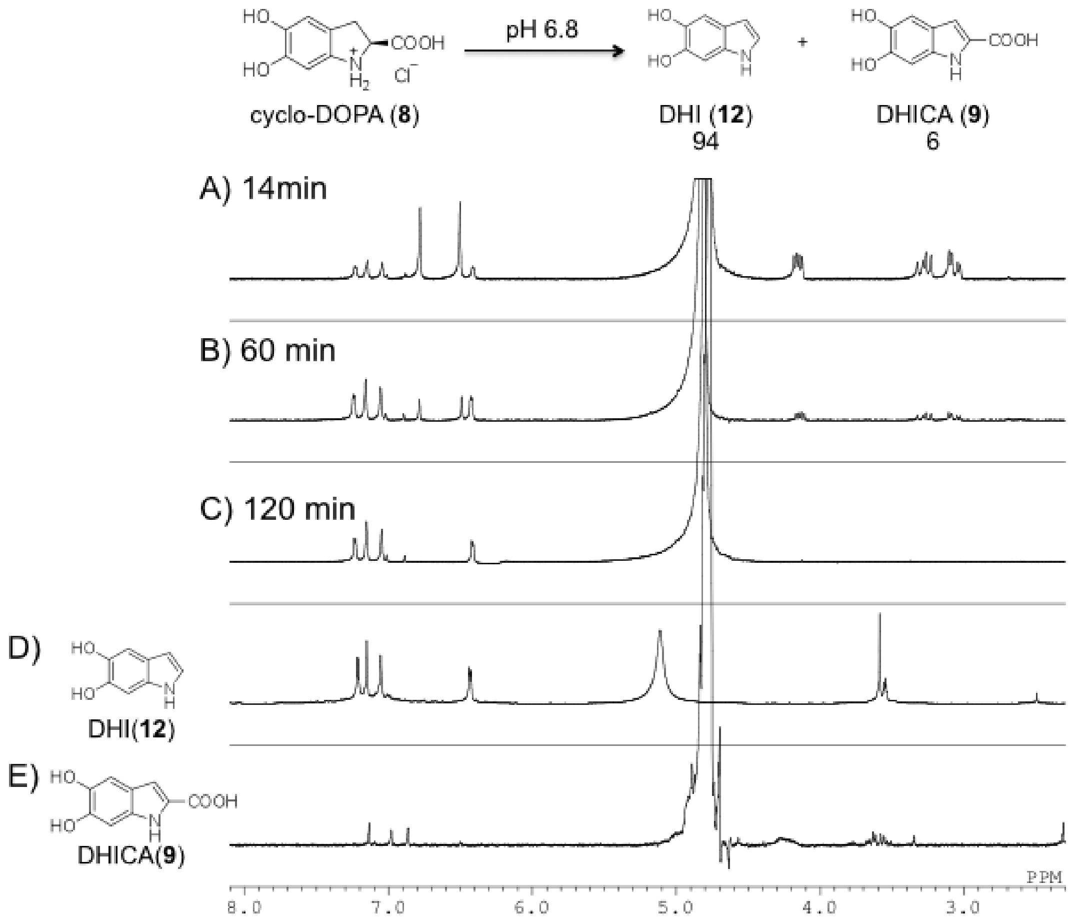

CycloDOPA (**8**) in D_2O (pH 1), pH of D_2O solution was adjusted to pH 6.8 with NaOD/D_2O and NMR spectrum was measured after 14, 60 and 120 min for A), B) and C), respectively. D) and E) are NMR spectrum for DHI (**12**) and DHICA (9), respectively

SM-4) Time course analysis for DPPH radical scavenge activity for cycloDOPA and its derivatives at pH 4 and 6.

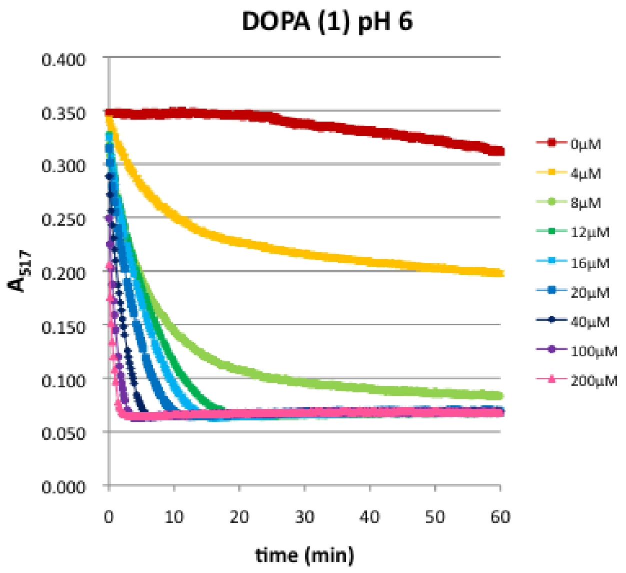

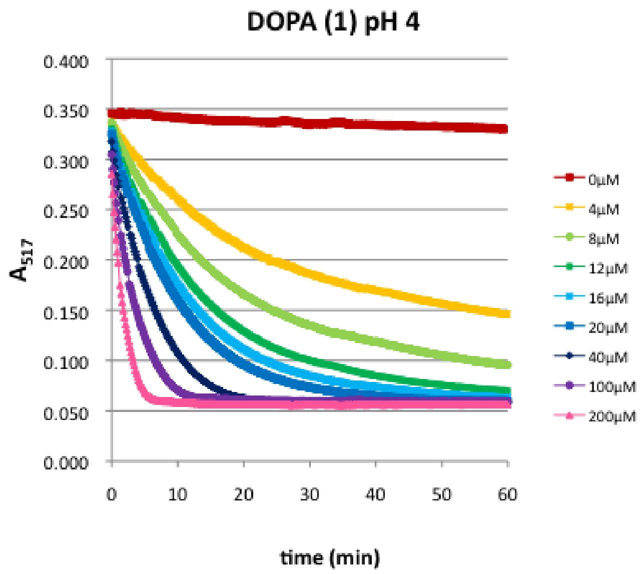

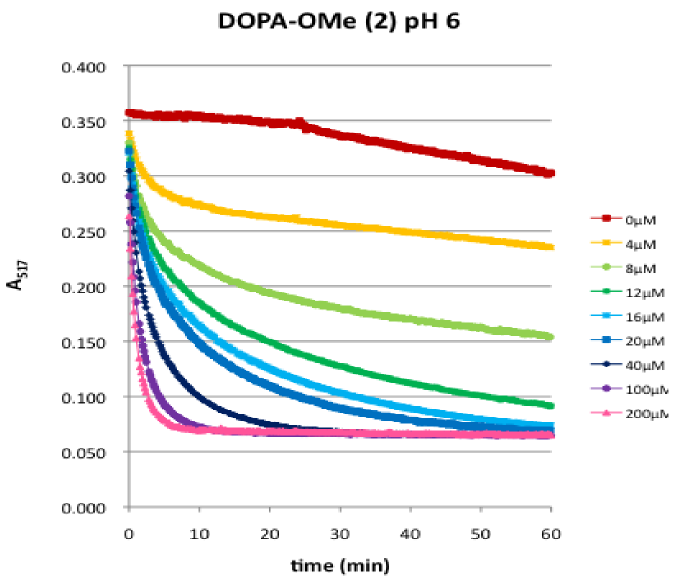

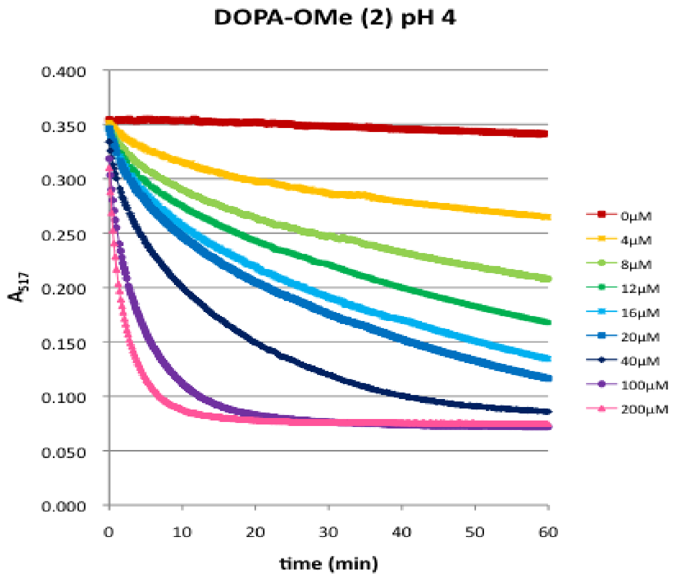

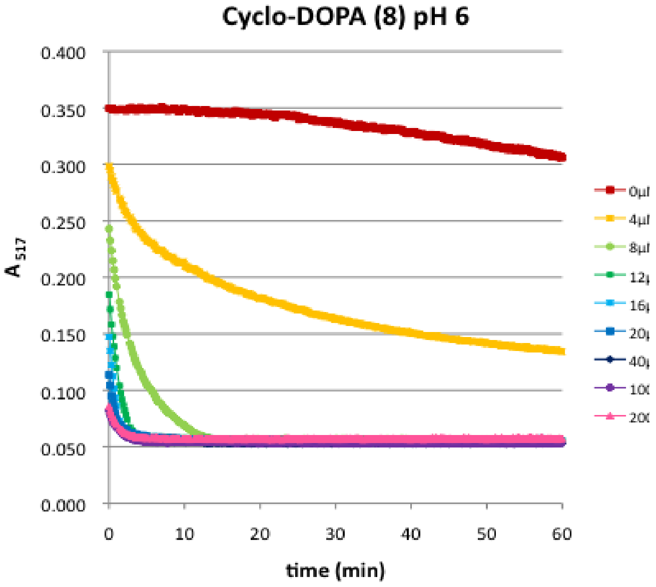
Cyclo-DOPA (8) pH 6
A 517
time (min)
0.400
0.350
0.300
0.250
0.200
0.150
0.100
0.050
0.000
0
10
20
30
40
50
60

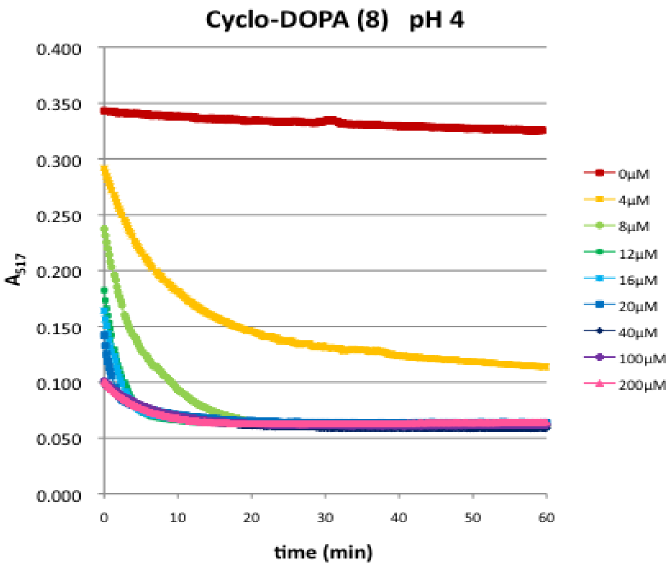
Cyclo-DOPA (8) pH 4
A_{517}
time (min)
0.400
0.350
0.300
0.250
0.200
0.150
0.100
0.050
0.000
0
10
20
30
40
50
60
0µM
4µM
8µM
12µM
16µM
20µM
40µM
100µM
200µM

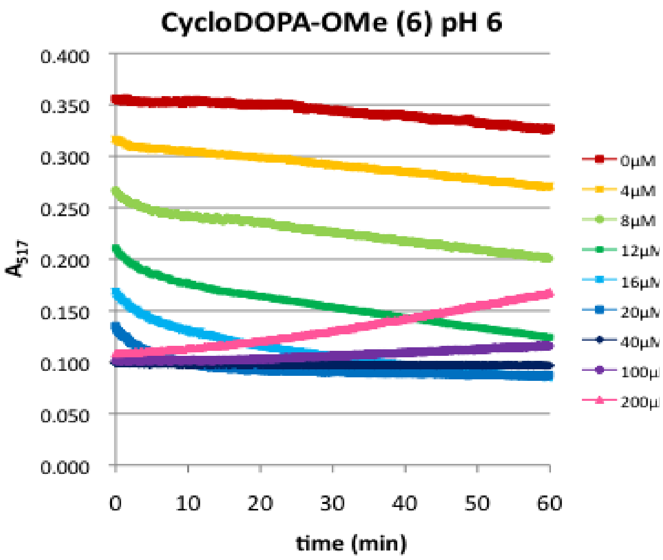
CycloDOPA-OMe (6) pH 6
A_{517}
time (min)
0.400
0.350
0.300
0.250
0.200
0.150
0.100
0.050
0.000
0
10
20
30
40
50
60
0µM
4µM
8µM

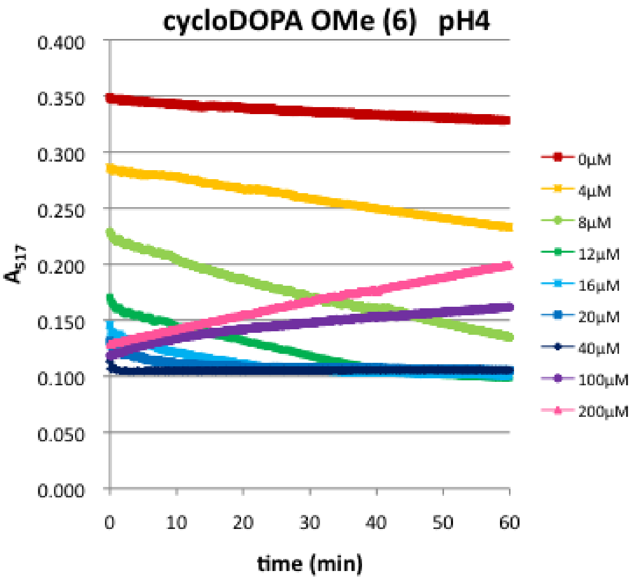
cycloDOPA OMe (6) pH4
A_{517}
time (min)
0.400
0.350
0.300
0.250
0.200
0.150
0.100
0.050
0.000
0
10
20
30
40
50
60
0µM
4µM
8µM
12µM
16µM
20µM
40µM
100µM
200µM

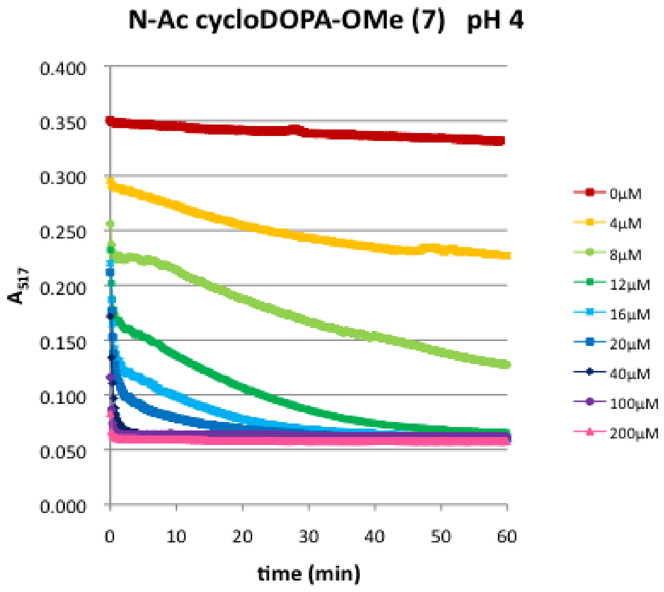
N-Ac cycloDOPA-OMe (7) pH 4
A_{517}
time (min)
0.400
0.350
0.300
0.250
0.200
0.150
0.100
0.050
0.000
0
10
20
30
40
50
60
0µM
4µM
8µM
12µM
16µM
20µM
40µM
100µM
200µM

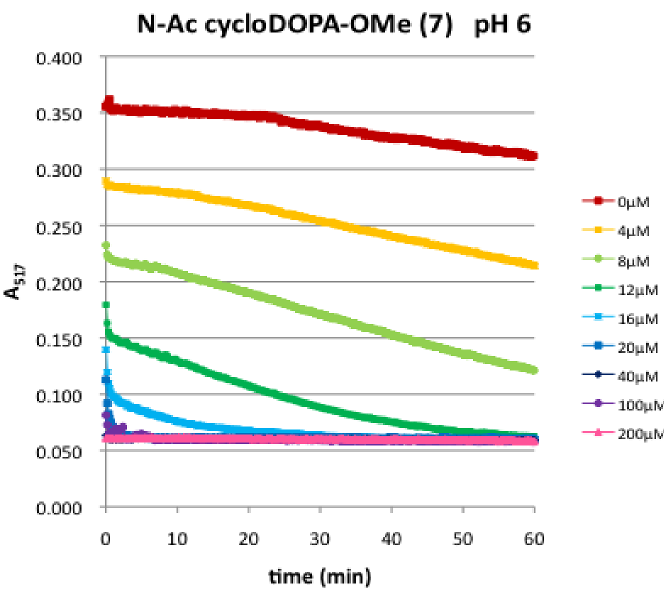
N-Ac cycloDOPA-OMe (7) pH 6
A_{517}
time (min)
0.400
0.350
0.300
0.250
0.200
0.150
0.100
0.050
0.000
0
10
20
30
40
50
60
0µM
4µM
8µM
12µM
16µM
20µM
40µM
100µ
200µ

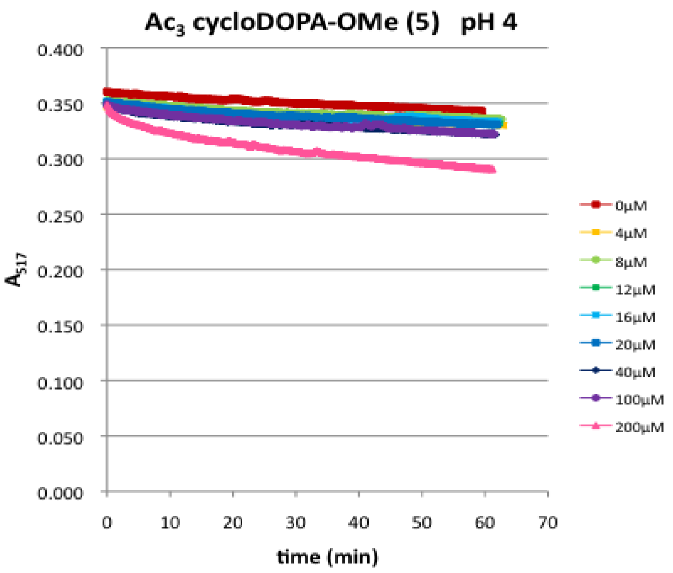
Ac_3 cycloDOPA-OMe (5) pH 4
A_{517}
time (min)
0.400
0.350
0.300
0.250
0.200
0.150
0.100
0.050
0.000
0
10
20
30
40
50
60
70
0µM
4µM
8µM
12µM
16µM
20µM
40µM
100µM
200µM

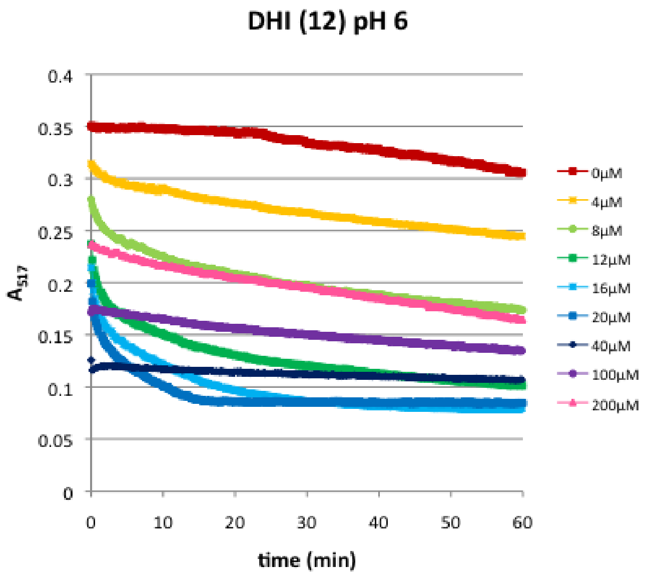
DHI (12) pH 6
A_{517}
time (min)
0.4
0.35
0.3
0.25
0.2
0.15
0.1
0.05
0
0
10
20
30
40
50
60
0µM
4µM
8µM
12µM
16µM
20µM
40µM
100µM
200µM

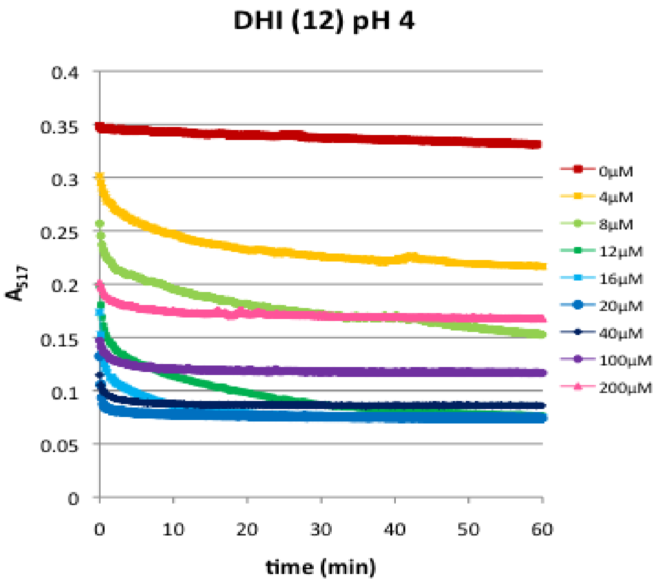
DHI (12) pH 4
A_{517}
time (min)
0.4
0.35
0.3
0.25
0.2
0.15
0.1
0.05
0
0
10
20
30
40
50
60
0µM
4µM
8µM
12µM
16µM
20µM
40µM
100µM
200µM

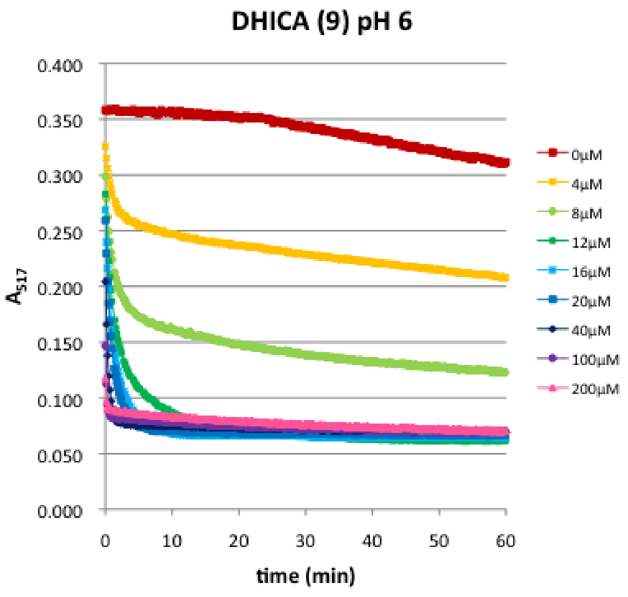
DHICA (9) pH 6
A_{517}
time (min)
0.400
0.350
0.300
0.250
0.200
0.150
0.100
0.050
0.000
0
10
20
30
40
50
60
0µM
4µM
8µM
12µM
16µM
20µM
40µM
100µM
200µM

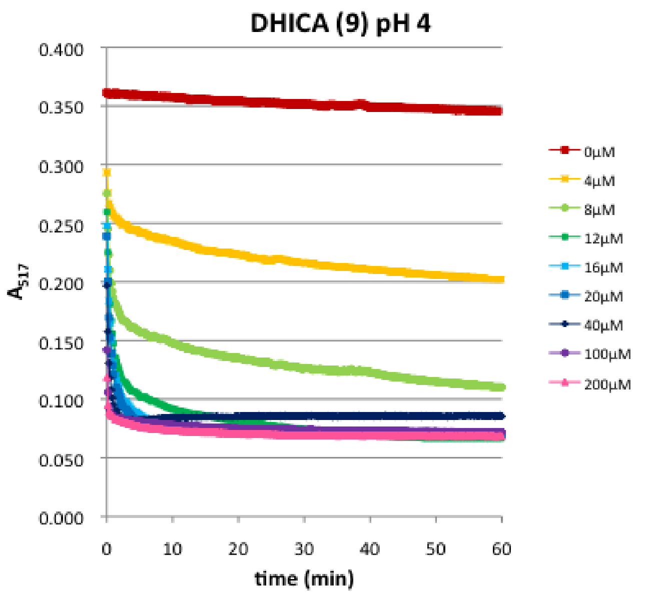
DHICA (9) pH 4
A_{517}
time (min)
0.400
0.350
0.300
0.250
0.200
0.150
0.100
0.050
0.000
0
10
20
30
40
50
60
0µM
4µM
8µM
12µM
16µM
20µM
40µM
100µM
200µM

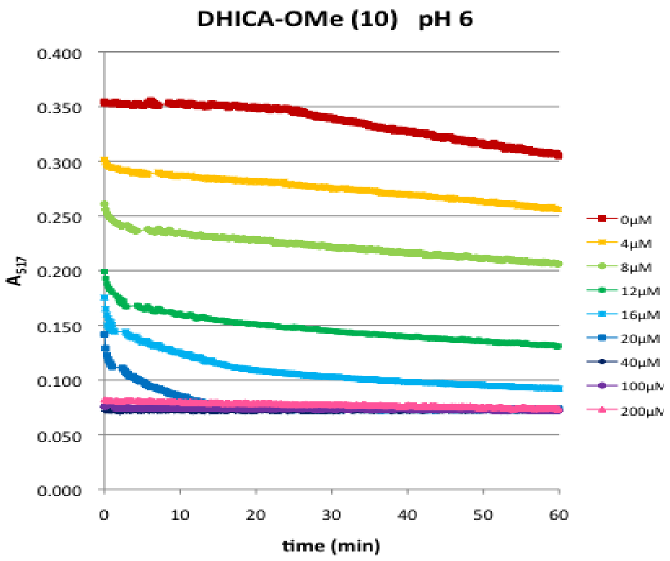
DHICA-OMe (10) pH 6
A517
time (min)
0µM
4µM
8µM
12µM
16µM
20µM
40µM

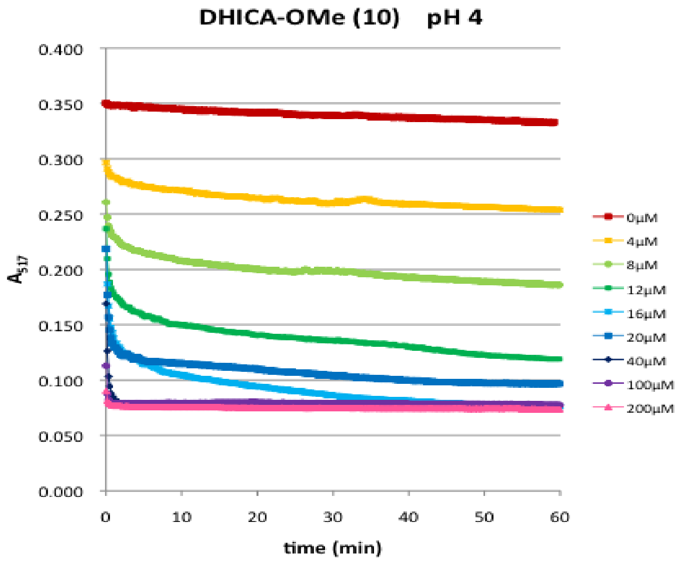
DHICA-OMe (10) pH 4
A517
time (min)
0µM
4µM
8µM
12µM
16µM
20µM
40µM
100µM
200µM

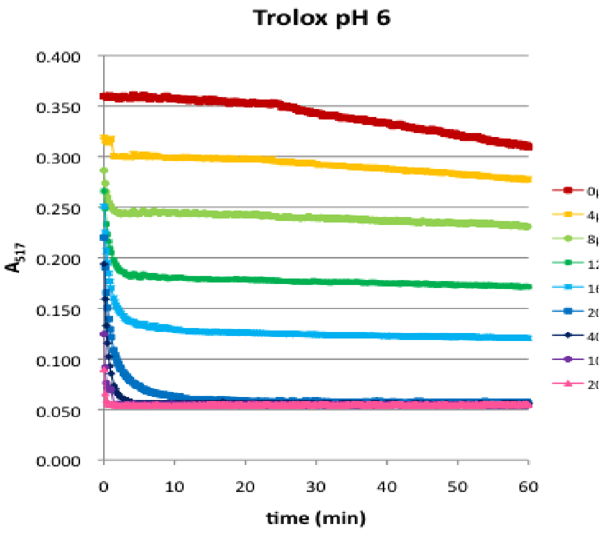
Trolox pH 6
A517
time (min)

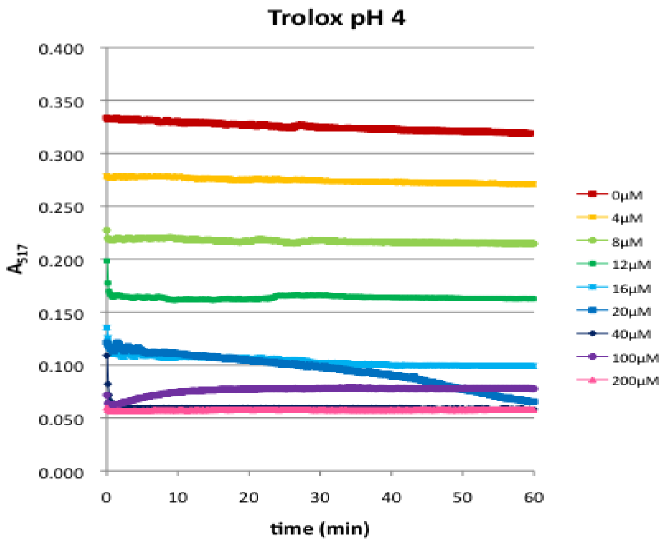
Trolox pH 4
A517
time (min)
0µM
4µM
8µM
12µM
16µM
20µM
40µM
100µM
200µM
